# Genetic determinants of genus-level glycan diversity in a bacterial protein glycosylation system

**DOI:** 10.1371/journal.pgen.1008532

**Published:** 2019-12-23

**Authors:** Chris Hadjineophytou, Jan Haug Anonsen, Nelson Wang, Kevin C. Ma, Raimonda Viburiene, Åshild Vik, Odile B. Harrison, Martin C. J. Maiden, Yonatan H. Grad, Michael Koomey

**Affiliations:** 1 Department of Biosciences, Center for Integrative Microbial Evolution, University of Oslo, Oslo, Norway; 2 Department of Immunology and Infectious Diseases, Harvard T. H. Chan School of Public Health, Boston, Massachusetts, United States of America; 3 Department of Zoology, University of Oxford, Oxford, United Kingdom; 4 Division of Infectious Diseases, Brigham and Women's Hospital and Harvard Medical School, Boston, Massachusetts, United States of America; 5 Centre for Ecological and Evolutionary Synthesis, University of Oslo, Oslo, Norway; Universidad de Sevilla, SPAIN

## Abstract

The human pathogens *N*. *gonorrhoeae* and *N*. *meningitidis* display robust intra- and interstrain glycan diversity associated with their *O*-linked protein glycosylation (*pgl*) systems. In an effort to better understand the evolution and function of protein glycosylation operating there, we aimed to determine if other human-restricted, *Neisseria* species similarly glycosylate proteins and if so, to assess the levels of glycoform diversity. Comparative genomics revealed the conservation of a subset of genes minimally required for *O*-linked protein glycosylation glycan and established those *pgl* genes as core genome constituents of the genus. In conjunction with mass spectrometric–based glycan phenotyping, we found that extant glycoform repertoires in *N*. *gonorrhoeae*, *N*. *meningitidis* and the closely related species *N*. *polysaccharea* and *N*. *lactamica* reflect the functional replacement of a progenitor glycan biosynthetic pathway. This replacement involved loss of *pgl* gene components of the primordial pathway coincident with the acquisition of two exogenous glycosyltransferase genes. Critical to this discovery was the identification of a ubiquitous but previously unrecognized glycosyltransferase gene (*pglP*) that has uniquely undergone parallel but independent pseudogenization in *N*. *gonorrhoeae* and *N*. *meningitidis*. We suggest that the pseudogenization events are driven by processes of compositional epistasis leading to gene decay. Additionally, we documented instances where inter-species recombination influences *pgl* gene status and creates discordant genetic interactions due ostensibly to the multi-locus nature of *pgl* gene networks. In summary, these findings provide a novel perspective on the evolution of protein glycosylation systems and identify phylogenetically informative, genetic differences associated with *Neisseria* species.

## Author summary

Bacteria express a remarkable diversity of sugars and oligosaccharides in conjunction with protein glycosylation systems. Currently however, little is known about the evolutionary processes and selective forces shaping glycan biosynthetic pathways. The closely related bacterial pathogens *Neisseria gonorrhoeae* and *Neisseria meningitidis* remain serious sources of human disease and these species express antigenically variable oligosaccharides as components of their broad-spectrum, *O*‐linked protein glycosylation (*pgl*) systems. With the exception of isolates of *Neisseria elongata* subspecies *glycolytica*, the status of such post-translational modifications in related commensal species colonizing humans remains largely undefined. Here, we exploit new data from further studies of protein glycosylation in *Neisseria elongata* subspecies *glycolytica* to address these concerns. Employing comparative genomics and glycan phenotyping, we show that related *pgl* systems are indeed expressed by all human-restricted *Neisseria* species but identify unique gene gain and loss events as well as loss-of-function polymorphisms that accommodate a dramatic shift in glycoform structure occurring across the genus. These findings constitute novel perspectives on both the evolution of protein glycosylation systems in general and the macroevolutionary processes occurring in related bacterial species residing within a single host.

## Introduction

Bacterial cell surfaces are decorated by diverse oligosaccharides and glycans in the context of capsules, lipopolysaccharides (LPS), glycoproteins and cell wall–associated glycoconjugates. Despite their ubiquity and implicit importance, the evolutionary processes shaping glycan diversity are not fully understood [1]. Such efforts are challenging as oligo- and polysaccharides are generated by the coordinated action of enzymes utilizing diverse monosaccharides and as specific functions of biosynthetic components and the glycans themselves are often undefined. For capsular polysaccharides and LPS, biosynthetic pathways are typically encoded within contiguous gene clusters. This linkage arrangement maintains biosynthetic compatibility allowing wholesale switching via single locus recombination events [2, 3]. Questions of the evolutionary processes and adaptive potential of glycans also apply to bacterial protein glycosylation systems in both their *N*- and *O*-linked forms [4]. Although both dedicated and broad-spectrum protein glycosylation are well recognized amongst eubacteria, relatively few studies have comprehensively examined glycan diversity and genotype–phenotype relationships at the genus level [5–8].

The genus *Neisseria* includes Gram-negative, oxidase-positive bacterial species that are associated with mucosal surfaces of humans and two closely related species are significant human pathogens. *Neisseria gonorrhoeae* is the agent of the sexually transmitted disease gonorrhea and *Neisseria meningitidis* is primarily a commensal of the oropharynx that under poorly understood circumstances can lead to invasive disease including meningitis. Despite their differing ecology and mechanisms of transmission, these species display remarkable conservation at the levels of nucleotide sequence, gene content and synteny [9]. Attempts to reconcile the distinctive relationships operating in these species with gene content are further complicated by the likewise, closely related species *N*. *lactamica and N*. *polysaccharea* that are harmless commensals found predominantly in the upper respiratory tracts of infants and children [10]. The genus also includes other less closely related nonpathogenic species that colonize the human oral cavity. Cross-species comparisons of genome sequences are beginning to reveal differences in gene content and organization and provide insights into evolutionary processes operating within the genus. Early studies using limited number of genomes or microarray-based genome hybridization studies concluded that a large number of “virulence” genes were distributed throughout the genus [11–13]. While studies of single gene families may be phylogenetically informative [9], analyses of multiple genes whose products function in concerted biosynthetic and biochemical pathways may be particularly resourceful. Recent examples of this include genus–wide analyses of genes involved in pilus biogenesis [11], determining cell shape (rod to coccus transitions) [14], protein glycosylation [11, 15], cytochrome c-based, electron transfer supporting dissimilatory nitrite reduction [16] and capsular polysaccharide expression [17].

Broad–spectrum, *O*-linked protein glycosylation (*pgl*) systems have been defined in *N*. *gonorrhoeae*, *N*. *meningitidis* and the deeply branching commensal species *N*. *elongata* subspecies *glycolytica*. Based on biochemical and reverse genetic approaches in tandem with mass spectrometry and serotyping for glycan characterization, consensus models for neisserial *pgl*-dependent protein glycosylation has been identified [15, 18–21]. A NAD^+^-dependent dehydratase (PglC) and aminotransferase (PglD) generate UDP-2-acetamido-4-amino-2,4,6-trideoxy-α-d-glucose from UDP-GlcNAc [20]. A bifunctional enzyme (PglB) then catalyzes amino acetylation of UDP-2-acetamido-4-amino-2,4,6-trideoxy-α-d-glucose to form UDP-di-*N*-acetylbacillosamine (diNAcBac) and the subsequent transfer of the phosphosugar to the lipid carrier undecaprenyl phosphate (Und-P) [20]. PglB2, encoded by *pglB2* alleles found in some *N*. *meningitidis* strains, contains a distinct *C*-terminal domain proposed to mediate the transfer of a glycerol moiety (in place of the acetyl group) to produce 4-glyceramido-2-acetamido-2,4,6-trideoxy-α-d-hexose (GATDH) [22]. Subsequent elaboration of these undecaprenyl diphosphate (Und-PP) monosaccharides ensues via two pathways using distinct glycosyltransferases. One involves PglH or its allelic variant-encoded PglH2, which attach a Glc or GlcNAc respectively, to the Und-PP-monosaccharides to generate disaccharides [5, 15]. The second pathway utilizes the PglA and PglE glycosyltransferases to add successive Gal units to produce a trisaccharide [21, 23]. As both pathways are active in some *N*. *gonorrhoeae* and *N*. *meningitidis* isolates, those strains can express simultaneously PglA- and PglH- generated glycoforms[15]. Moreover, PglH/PglH2-generated Und-PP-disaccharides are incapable of being further extended by PglE [5, 15]. Antagonism and potential redundancy involving PglA and PglH in *N*. *gonorrhoeae* and *N*. *meningitidis* have been hypothesized to account for hypomorphic *pglA* and *pglH* alleles as well as a *pglH* deletion mutation found in some strains of the two species [15, 24]. Studies in *N*. *elongata* subspecies *glycolytica* (that lack the *pglA* and *pglE* genes) reported the expression of a di-*N*-acetylbacillosamine-glucose-di-*N*-acetyl glucuronic acid-*N*-acetylhexosamine (diNAcBac-Glc-diNAcHexA-HexNAc) tetrasaccharide [25]. There, the addition of the diNAcHexA moiety at the third position (onto a PglB,C,D and H-dependent Und-PP-diNAcBac-Glc disaccharide) was shown by mutagenesis to require the *pglG* gene whose product is predicted to be a glycosyltransferase. It also required four genes (*pglJ*, *K*, *M* and *N*) whose products operate in the step-wise synthesis of the UDP-diNAcGlcA donor [26]. Interestingly, orthologues of *pglG* are found in most strains of in *N*. *gonorrhoeae* and *N*. *meningitidis* (where they map just upstream of *pglH*/*H2*) but there is no evidence to date there that *pglG* impacts on glycoform phenotype in those backgrounds [5, 15, 18, 23, 27]. The potential distribution of *pglJ*, *K*, *M* and *N* gene orthologues in *N*. *gonorrhoeae* and *N*. *meningitidis* has not been reported.

Another feature distinguishing the *pgl* systems of *N*. *gonorrhoeae* and *N*. *meningitidis* from that of *N*. *elongata* subspecies *glycolytica* is their abilities to undergo high frequency, intrastrain glycoform antigenic variation. This phenomenon results from the presence of hypermutable, simple nucleotide repeat elements mapping within the ORFs of the *pglA*, *pglE* and *pglH* glycosyltransferase genes [5, 15, 18, 22, 23, 28, 29]. Stochastic changes in nucleotide repeat copy number there result in on-off glycosyltransferase expression with corresponding alterations in glycoform expression. Such repeat elements are not recognizable in *pgl* genes from *N*. *elongata* subspecies *glycolytica*. These data combined with the fact that neisserial protein-associated glycoforms possess unique immunogenic and antigenic properties [19] strongly suggests that *pgl* glycoforms in *N*. *gonorrhoeae* and *N*. *meningitidis* are subject to diversifying selection [5, 15, 18, 19, 22, 30–32].

In *N*. *gonorrhoeae* and *N*. *meningitidis*, the most abundant glycoproteins are the PilE pilin proteins which are the major subunit of their type IV pilus colonization (Tfp) factors [29, 33, 34]. Tfp are primary mediators of adherence to human epithelial cells [35, 36] and are required for persistence and disease in experimental gonococcal infection of human male volunteers [37]. Analogous roles for meningococcal Tfp are predicted. The glycosylation status of PilE has been linked with alterations in Tfp-associated phenotypes including autoagglutination, dynamics of organelle extrusion-retraction, adherence to human cells and the proficiency of pilin polymerization [32, 38–40]. Moreover, the glycans of PilE are oriented in a fashion such that they are exposed on the surface of intact Tfp. Furthermore, the PilE subunit protein is subject to extensive antigenic variation (changes in primary structure) in gonococci and a subset of meningococcal strains due to gene conversion-like events between partial, truncated donor alleles and an active expression locus [41]. Thus, PilE glycoproteins are subject to two levels of intrastrain structural variability: one at the level of the protein itself and the other at the level of the attached glycan. PilE intrastrain diversity further complicates attempts to define glycan function as it remains unclear if the effects of glycosylation on Tfp phenotypes are broadly applicable or variant PilE-specific. In *N*. *elongata* subspecies *glycolytica*, PilE is neither subject to antigenic variation nor glycosylated [25].

The complexity and variability of protein glycosylation in this genus and the commonalities of glycosylation in the two pathogenic species prompted us to determine if other human—restricted, *Neisseria* species similarly glycosylate proteins and if so, to assess the genotype–phenotype relationships acting there. Using comparative genomics and mass spectrometric (MS)-based glycan phenotyping, we identify here gene loss events and loss-of-function polymorphisms at multiple loci that accommodate a shift in glycoform structure occurring across the genus. Using this *pgl*-centric approach, we also present compelling examples as to how recombination can both reverse epistasis—associated gene inactivation as well as create seemingly discordant gene networks.

## Results

### Identification of a glycosyltransferase required for *N*. *elongata* subsp. *glycolytica* tetrasaccharide synthesis

**Fig 1:**
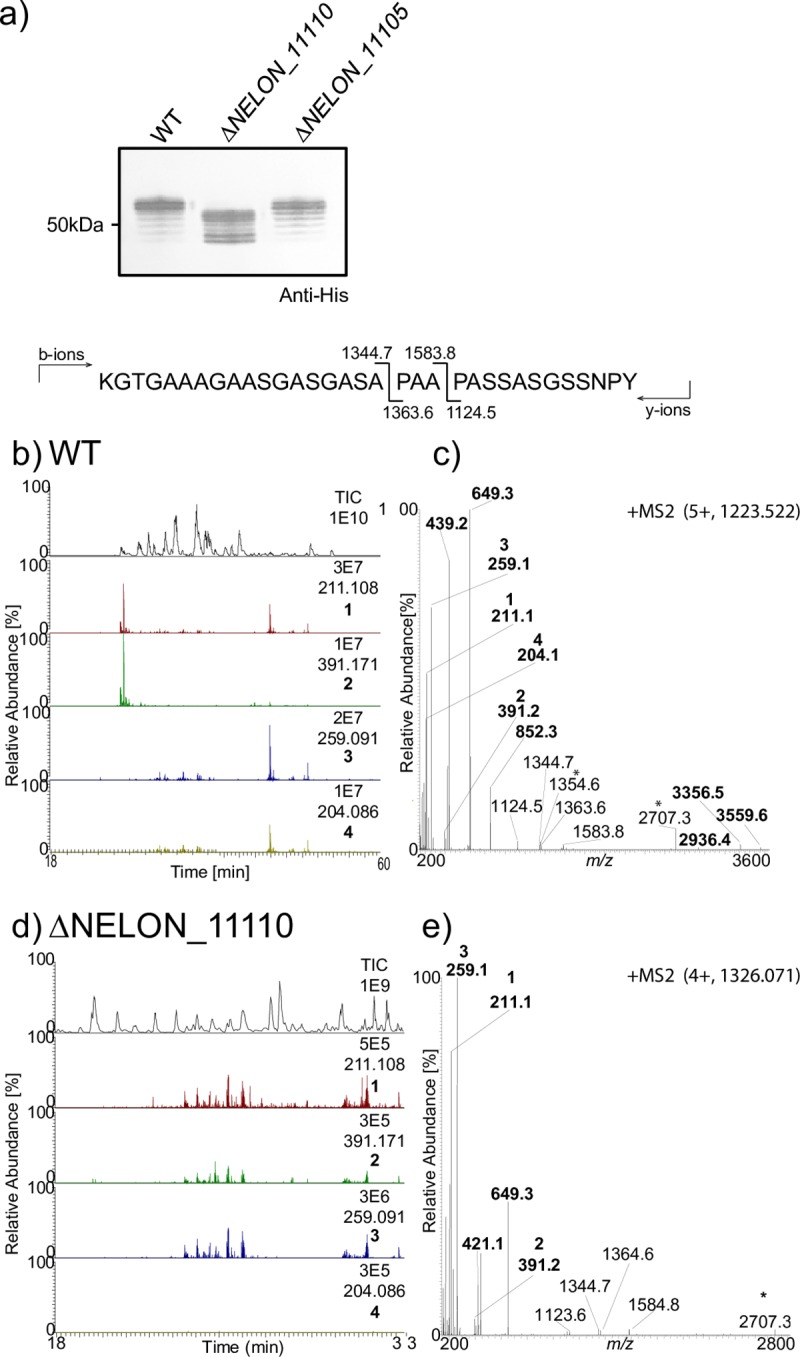
Identification of NELON_1110 as a *pgl* glycosyltransferase. a) Immunoblot of whole-cell lysates from strains expressing NirK-His6x using the wild type (WT, KS992), ΔNELON_11110 (KS1032) and ΔNELON_11105 (NW270) strains (S1 Fig, S1 Table) using a tetra-His epitope recognizing antibody. Multiple isoforms of NirK-His are the result of macroheterogeneity (variable glycan site occupancy) as NirK has five potential sites of glycan addition. b-e) Liquid chromatography tandem MS (LC-MS2) chromatograms of the peptide shown from affinity purified NirK from WT and a ΔNELON_11110 mutant. Total ion chromatogram (TIC) intensity values represents amounts of peptides entering the mass spectrometer. The selected ion chromatograms (SIC) are of the four glycan reporter ions characteristic for a tetrasaccharide; diNAcBac at *m/z* 211.108 (1), diNAcBac-Hex at *m/z* 391.170 (2), diNAcHexA at *m/z* 259.093 (3) and HexNAc at *m/z* 204.086 (4). The MS2 spectrum demonstrates the presence of glycan reporter ions (marked in bold and numbered as defined above). b) The LC-MS2 chromatogram of the NirK derived peptide from a WT background. c) MS2 spectrum of the peptide from a WT (in panel b) background bearing a diNacBac-Hex-diNAcHexA-HexNAc tetrasaccharide. d) The LC-MS2 chromatogram the peptide from the ΔNELON_11110 background. e) MS2 spectrum of the peptide ΔNELON_11110 background (panel d) carrying the diNacBac-Hex-diNAcHexA trisaccharide.

**Fig 2:**
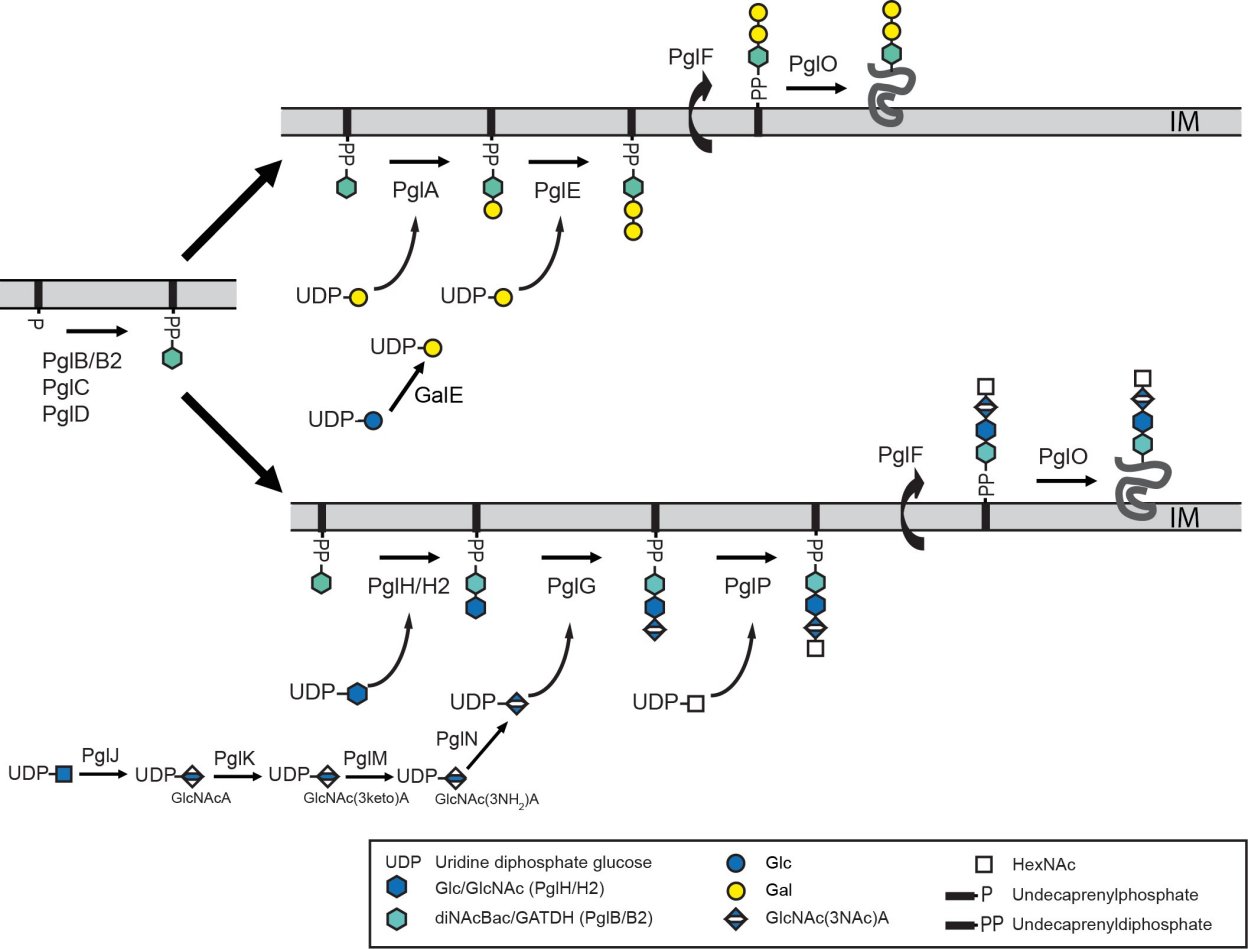
Divergent *pgl* pathways for glycan biosynthesis defined in *N*. *gonorrhoeae*, *N*. *meningitidis* and *N*. *elongata* subsp. *glycolytica*. The two non-interactive pathways are defined as utilizing the products of the *pglA/E* genes (top) or the *pglG/H/P* genes (bottom). The *pglA/E* pathway requires the product of *galE* to provide UDP–galactose while the *pglG/H/P* pathway is absolutely dependent on *pglJ* (but not *pglK*, *pglM* or *pglN*) to provide UDP–glucuronic acid. (See [26] and text for further details).

To perform a comprehensive comparative analysis of *pgl* genes in *Neisseria*, we first determined the complete glycan synthesis pathway of *N*. *elongata* subsp. *glycolytica*. Studies of *N*. *elongata* subsp. *glycolytica* revealed a tetrasaccharide glycoform comprised of di-*N*-acetylbacillosamine–glucose–di-*N*-acetylglucuronic acid and *N*-acetylhexosamine [diNAcBac-Glc-HexNAc(3NAc)A-HexNAc] [25]. There, mutagenesis and glycan profiling defined the role of *pglH* encoding the glycosyltransferase incorporating glucose at the second position to create a disaccharide (as it does in *N*. *gonorrhoeae* and *N*. *meningitidis* [5, 15]). This structure was further extended by the PglG glycosyltransferase to generate a diNAcBac-Glc- HexNAc(3NAc)A acid trisaccharide. Synthesis of the UDP–di-*N*-acetylglucuronic acid precursor entailed the sequential activities of four *N*. *elongata* subsp. *glycolytica* enzymes (encoded by *pglJ*, *K*, *M* and *N*) starting with UDP-GlcNAc [26]. That work did not identify the glycosyltransferase responsible for addition of the terminating HexNAc moiety. We screened *pgl* gene clusters in genomes of species closely related to *N*. *elongata* subsp. *glycolytica* to identify potential glycosyltransferases. There, an ORF predicted to encode a glycosyltransferase with an *N*-terminal glycosyltransferase family 4 domain (PF13439) and a *C*-terminal glycosyltransferase group 1 domain (PF00534) was identified in an *N*. *oralis* strain. Using that nucleotide sequence, queries of *N*. *elongata* subsp. *glycolytica* genomes yielded an ORF sharing significant identity (designated NELON_11110) that mapped just 3´of a gene previously identified as a potential *pgl*-dedicated flippase (*pglF*-NELON_11115). Mutagenic disruption of the putative glycosyltransferase ORF led to increased mobility of the NirK glycoprotein in SDS-PAGE and MS-based analyses of purified NirK revealed the presence of a diNAcBac-Glc-HexNAc(3NAc)A trisaccharide (Fig 1). These altered phenotypes were not readily attributable to a polar effect of the mutation on distal gene expression as they were not seen following disruption of the downstream gene (NELON_11105) (Fig 1) nor in a NELON_11110 mutant carrying a short in-frame deletion (Fig 1, S1 Fig). We conclude that NELON_11110 encodes the glycosyltransferase responsible for synthesis of the mature *N*. *elongata* subsp. *glycolytica* tetrasaccharide and termed it *pglP*. A summary of the bifurcating pathways for glycan biosynthesis as defined here and in earlier studies is shown in Fig 2.

### Distribution of *pgl* genes across the genus *Neisseria*

**Fig 3:**
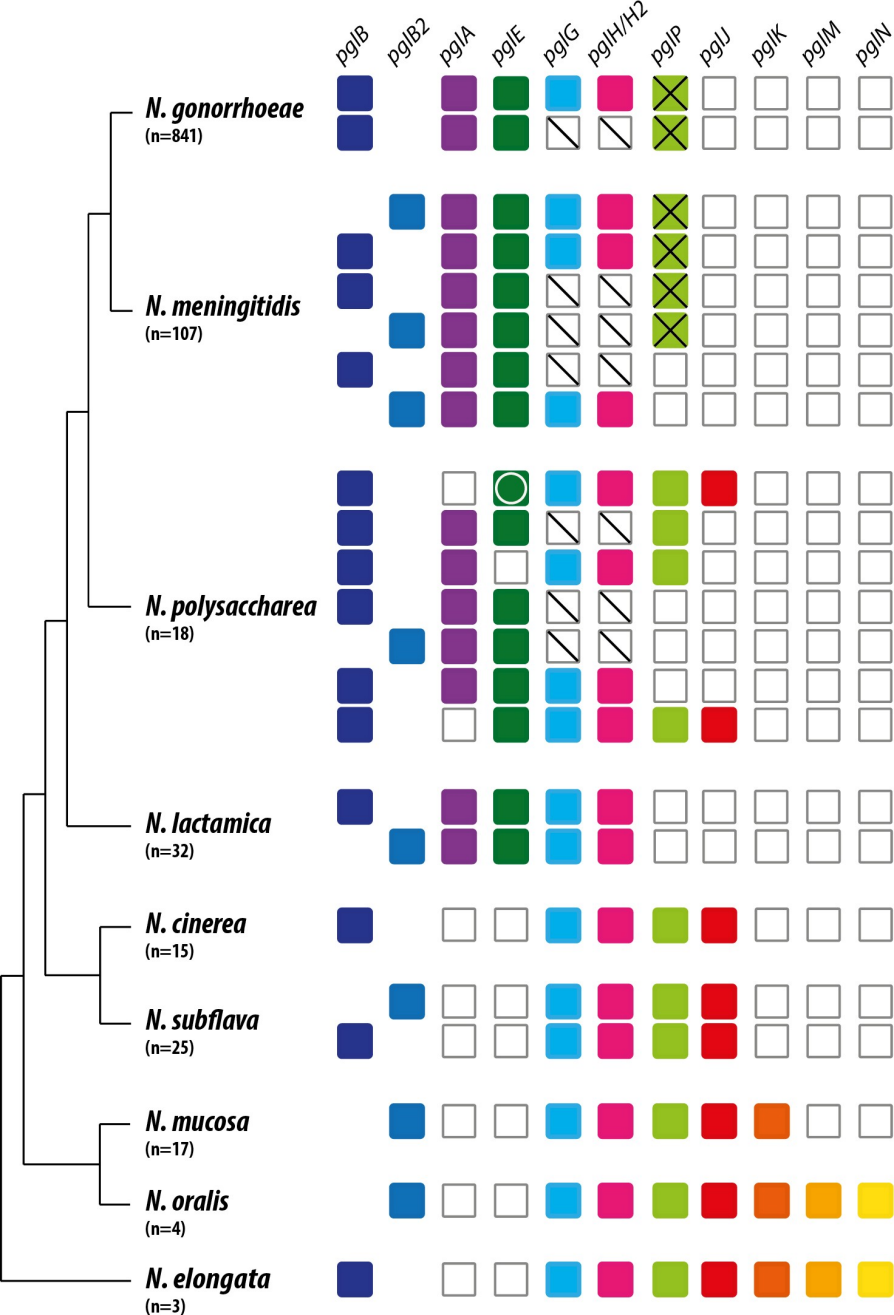
Presence and status of *pgl* genes shaping glycoform diversity in neisserial species groups. Color boxes indicate gene presence while white boxes indicate gene absence. Boxes with an X denote alleles with ORF-disrupting SNVs and/or CREE insertions while those for *pglG* and *pglH* with a diagonal line denote a conserved, inactivating deletion spanning the 3´end of *pglG* and the 5´end of *pglH*. The *pglE* gene with a white circle indicates alleles with the insertion of an IS element. The data are superimposed on a tree of species group relationships established using neighbour‐joining phylogeny (modified from that generated in [9]). n = number of strain genomes included. For each species group, patterns shown are ranked by relative abundance from highest (top) to lowest. Strains used are found in S1 Dataset. Note that *N*. *meningitidis* CC269 isolates bearing intact ORF *pglP* genes are not included here. Also, *N*. *meningitidis pglH* alleles carrying IS element insertions have been reported by others but such strains were not in the datasets used for this figure [32]. Neisseria PubMLST loci designations are found in S3 Table.

We analyzed the genomes of temporally and geographically distributed strains of human associated *Neisseria* species (S1 Dataset) for *pgl* gene content (Fig 3, n = number of species group genomes included). All genomes contained orthologues of the *pglB/pglB2, pglC* and *pglD*, the products of which act in the synthesis of uridine diphosphate (UDP)-sugar (PglD, PglC, and PglB-acetyltransferase domain) and the transfer of the phospho-sugar to undecaprenyl phosphate (Und-P) (PglB-phospho-glycosyltransferase domain [20, 21]). PglB catalyzes the synthesis of Und-PP-N’-diacetylbacillosamine (diNAcBac) while PglB2 catalyzes the synthesis of Und-PP-glyceramido-acetamido trideoxyhexose (GATDH) [20, 22]. Orthologues of the *pglG* and *pglH* genes were found in genomes from all human associated species across the genus and that were without exception, arrayed tandemly and mapped just upstream of the *pglB/B2*, *C*, and *D* genes (Fig 3 and S2 Fig). However, in a subset of *N*. *gonorrhoeae*, *N*. *meningitidis* and *N*. *polysaccharea* isolates there is a stereotypic deletion encompassing the 3´ end of *pglG* and the 5´ end segment of *pglH* (Fig 3 and S2 Fig). The deletion endpoint sequences in these mutants were highly conserved indicating the likely dissemination of a founder mutation across species by horizontal gene transfer (HGT). Orthologues of *pglF* (whose product is implicated in translocation of the Und-PP-oligosaccharides from a cytoplasmic to a periplasmic orientation [21] were identifiable as high-quality hits situated within 0.1–5 kB upstream of their *pglB/B2*, C and *D* genes. Thus, the synteny of the *pglF*, *pglG*, *pglH*, *pglB/B2*, *pglC* and *pglD* cluster was remarkably conserved across the genus with only limited exceptions involving the *pglG*/*H* deletion and a few interspersed ORFs seen in some deeply branching commensal genomes (S2 Fig). Accordingly, we termed this region the *pgl* core locus. All genomes also contained genes orthologous to *pglL (*also known as *pglO)* encoding protein–targeting oligosaccharyltransferases [21, 42] that in each instance mapped outside the core locus (S2 Fig).

In line with previous estimates, the *pglA* and *pglE* glycosyltransferase genes (unlinked to the core loci and one another) were found in all *N*. *gonorrhoeae*, *N*. *meningitidis* and *N*. *lactamica* strains and there was evident microsynteny conservation at both loci across these species (Fig 3, S2, S3 and S4 Figs). In contrast, these genes were absent from other commensal species save for *N*. *polysaccharea* where they were differentially distributed (Fig 3, S2, S3 and S4 Figs). The *pglA* and *pglE* genes were present in 61% and 55% of *N*. *polysaccharea* isolates respectively. In those *N*. *polysaccharea* strains bearing *pglE*, there was conserved microsynteny with equivalent loci in *N*. *gonorrhoeae*, *N*. *meningitidis* and *N*. *lactamica*. The *pglA* gene is absent in 39% of *N*. *polysaccharea* isolates while for *pglE*, strains carried an intact allele, an allele with an insertion of an IS element or lacked the gene altogether (Fig 3, S2, S3 and S4 Figs). In those *N*. *polysaccharea* strains bearing *pglE*, there was clear microsynteny conservation between the loci and those in *N*. *lactamica* and some *N*. *cinerea* isolates while in those lacking *pglE*, synteny was seen with the equivalent loci in some strains of *N*. *cinerea* (S4 Fig). A similar relationship was detected for *N*. *polysaccharea* strains lacking *pglA* where there was shared microsynteny with loci in strains of *N*. *cinerea* (S3 Fig).

Next, the status of *pglG* in *N*. *gonorrhoeae* and *N*. *meningitidis* was investigated. Given the requirement for a UDP-glucuronic acid donor for PglG to function (as defined in *N*. *elongata* subsp. *glycolytica* [25]), we assessed the genus-wide status of *pglJ* encoding a dehydrogenase essential to synthesis of the UDP-sugar from UDP-GlcNAcA. In contrast to the widespread presence of *pglG*, *pglJ* was absent from the genomes of *N*. *gonorrhoeae*, *N*. *meningitidis* and *N*. *lactamica* species, variably distributed in genomes of *N*. *polysaccharea* strains and present in all other commensal genomes examined (Fig 3). Similarly, *pglK* required for the second step in synthesis of UDP-di-*N*-acetylglucuronic acid from UDP-glucuronic acid was absent from *N*. *gonorrhoeae*, *N*. *meningitidis*, *N*. *polysaccharea* and *N*. *lactamica* genomes but present in those of strains of the *N*. *mucosa*, *N*. *oralis* and *N*. *elongata* species groups (the last three of which all possess the *pglJ—*encoding dehydrogenase). The *pglM* and *pglN* genes (acting downstream of *pglJ* and *pglK* Fig 2) were limited to *N*. *oralis* and *N*. *elongata* species groups (Fig 3). Therefore, the pathogenic species together with *N*. *polysaccharea* and *N*. *lactamica* appear to be incapable of synthesizing the UDP-sugar donor utilized by PglG.

### Analyses of protein glycan diversity in commensal neisserial species groups

**Fig 4:**
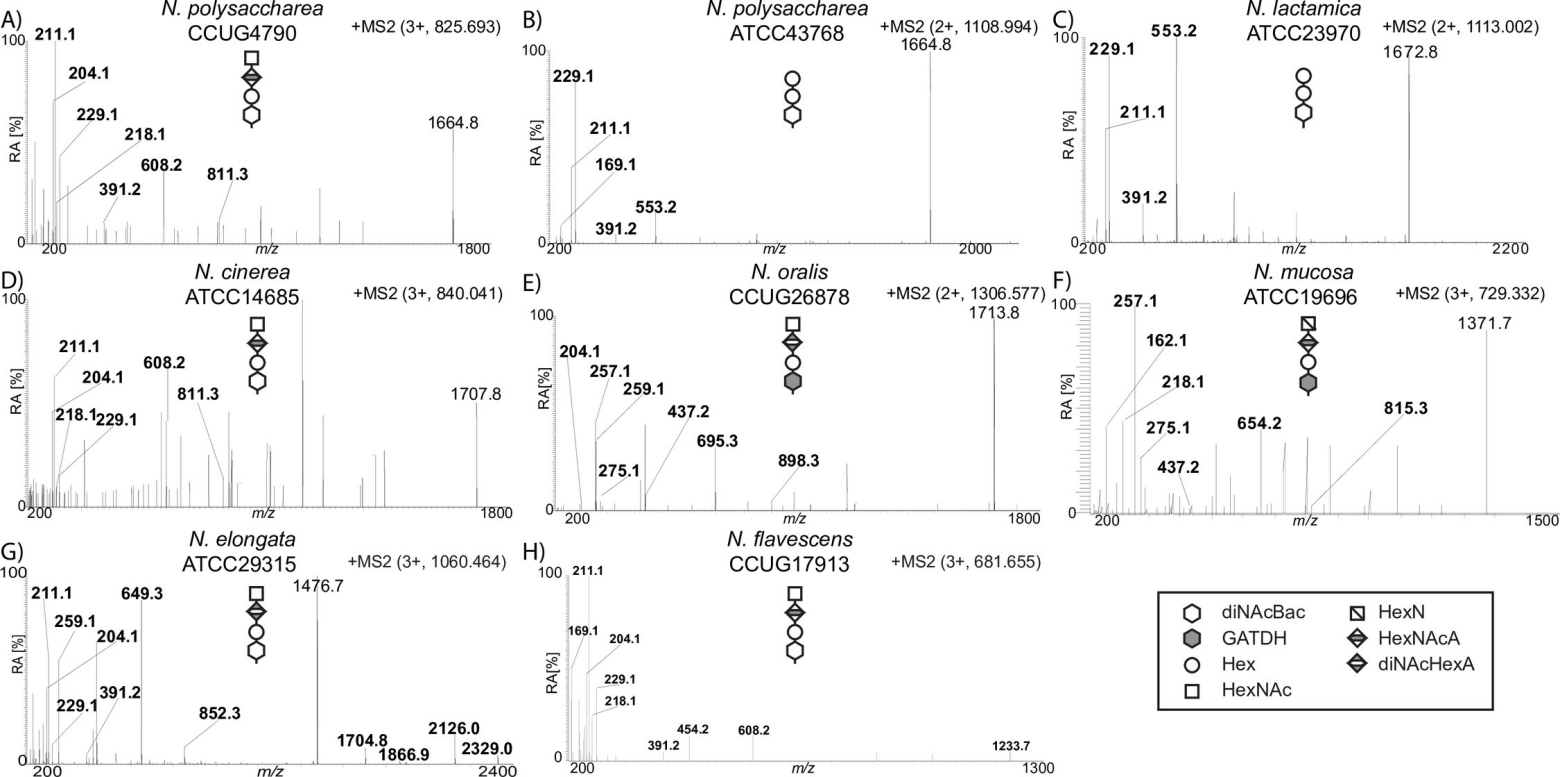
Targeted MS analysis of glycan structures shows presence of HexNAc/HexN incorporating glycoforms in select commensal strains. The reporter ions for the protein attached glycans are: diNAcBac at *m/z* 229.1/211.1, diNAcBac-Hex at *m/z* 391.2, diNAcBac-Hex-Hex at *m/z* 553.2, diNAcBac-Hex-HexNAcA at *m/z* 608.2, diNAcBac-Hex-diNAcHexA at *m/z* 649.3, diNAcBac-Hex-HexNAcA-HexNAc at *m/z* 811.3, diNAcBac-Hex-diNAcHexA-HexNAc at *m/z* 852.3. GATDH at *m/z* 275.1/257.1, GATDH-Hex at *m/z* 437.1, GATDH-Hex-HexNAcA at *m/z* 654.2, GATDH-Hex-diNAcHexA at *m/z* 695.3, GATDH-Hex-HexNAcA-HexN at *m/z* 815.3, GATDH-Hex-diNAcHexA-HexNAc at *m/z* 852.3, HexN at *m/z* 162.1, HexNAc at *m/z* 204.1, HexNAcA at *m/z* 218.1 and diNAcHexA at *m/z* 259.093. A) MS2 spectrum of a glycopeptide carrying a diNAcBac-Hex-HexNAcA-HexNAc from *N*. *polysaccharea* CCUG4790. B) MS2 spectrum of a glycopeptide carrying a diNAcBac-Hex-Hex from *N*. *polysaccharea* ATCC43768. C) MS2 spectrum of a glycopeptide carrying a diNAcBac-Hex-Hex from *N*. *lactamica* ATCC23970. D) MS2 spectrum of a glycopeptide carrying a diNAcBac-Hex-HexNAcA-HexNAc from *N*. *cinerea* ATCC14685. E) MS2 spectrum of a glycopeptide carrying a GATDH-Hex-diNAcHexA-HexNAc from *N*. *oralis* CCUG26878. F) MS2 spectrum of a glycopeptide carrying a GATDH-Hex-HexNAcA-HexN from *N*. *mucosa* ATCC19696. G) MS2 spectrum of a glycopeptide carrying a diNAcBac-Hex-diNAcHexA-HexNAc from *N*. *elongata* ATCC29315. H) MS2 spectrum of a glycopeptide carrying a diNAcBac-Hex-HexNAcA-HexNAc from *N*. *flavescens* CCUG17913. All strains used are species type strains.

**Fig 5:**
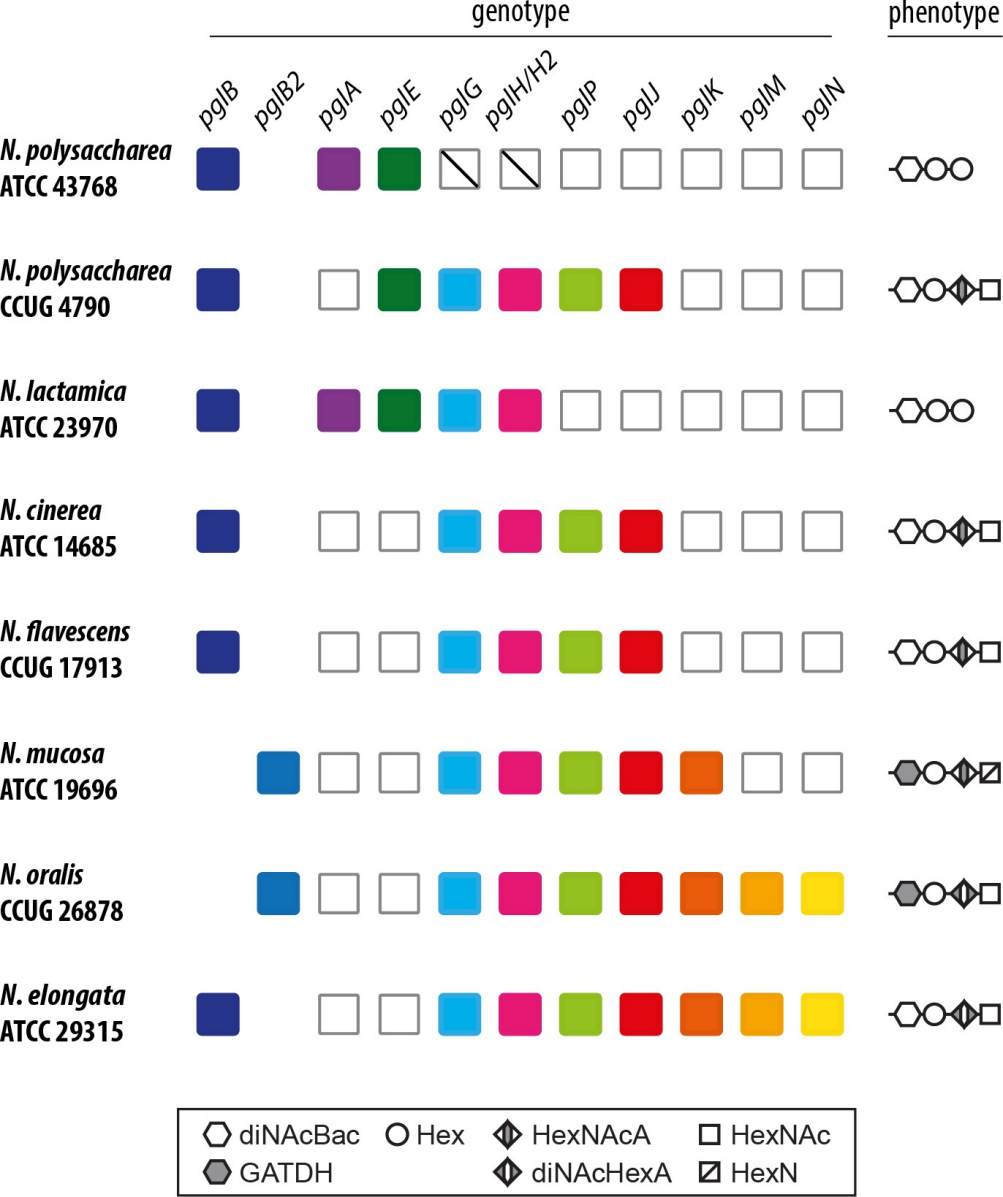
Genotype / phenotype relationships associated with glycan structures in select commensal strains. All strains used are species type strains. See Figs 3 and 4 for details.

We next sought to delineate the prevalence of protein glycosylation across neisserial species and to examine *pgl* genotype–phenotype relationships. As such connections were already defined for *N*. *gonorrhoeae*, *N*. *meningitidis*, and *N*. *elongata* subsp. *glycolytica*, focus was placed on remaining commensal species. Using a shot-gun MS approach, oligosaccharides composed of 3–4 sugar residues were identified from all isolates tested (Figs 4 and 5). While MS cannot define glycan stereochemistry, the detection of specific oxonium ions and related fragmentation products are diagnostic for particular sugars. Using this approach, correlations between *pgl* gene content and oligosaccharide structure were readily observed (Fig 5). For example, the incorporation of hexuronic acid or its modified derivatives at the third position was associated with the presence of *pglG*, *pglH* and *pglJ* while the presence of hexose at the third residue was associated with *pglA* and *pglE*. In addition, the presence of either HexNAc or HexN at the fourth position correlated with the presence of *pglP* together with *pglG*, *pglH* and *pglJ*. These correlations were emphasized by results for two strains of *N*. *polysaccharea* differing in *pgl* gene content and glycan structures (Figs 3, 4 and 5). These data reveal that protein glycosylation is manifest throughout the genus and establish a strong correspondence between *pgl* gene content and glycoform repertoires.

### Species-specific pseudogenization and gene loss of *pglP*

As *pglP* acts downstream of *pglG* and *pglJ* (as defined in *N*. *elongata* subsp. *glycolytica* [26]), it was of interest to assess its distribution across the genus. Alleles of *pglP* were identified within all species groups except *N*. *lactamica* and a subset of *N*. *meningitidis* and *N*. *polysaccharea* isolates (Fig 3, S2 and S5 Figs). In these latter instances, there was microsyntenic conservation at the associated loci across all 3 species (S5 Fig). Alleles from commensal species groups *N*. *elongata*, *N*. *oralis*, *N*. *mucosa*, *N subflava*, and *N*. *cinerea* and *N*. *polysaccharea* encoded intact ORFs whose corresponding polypeptides were highly related to one another (S6 and S7 Figs). To examine their functional conservation, allelic replacement was used to introduce representative *pglP* genes from *N*. *oralis* and *N*. *cinerea* into *N*. *elongata* subsp. *glycolytica* where complementation was observed by immunoblotting of the NirK glycoprotein (S1 Fig).

**Fig 6:**
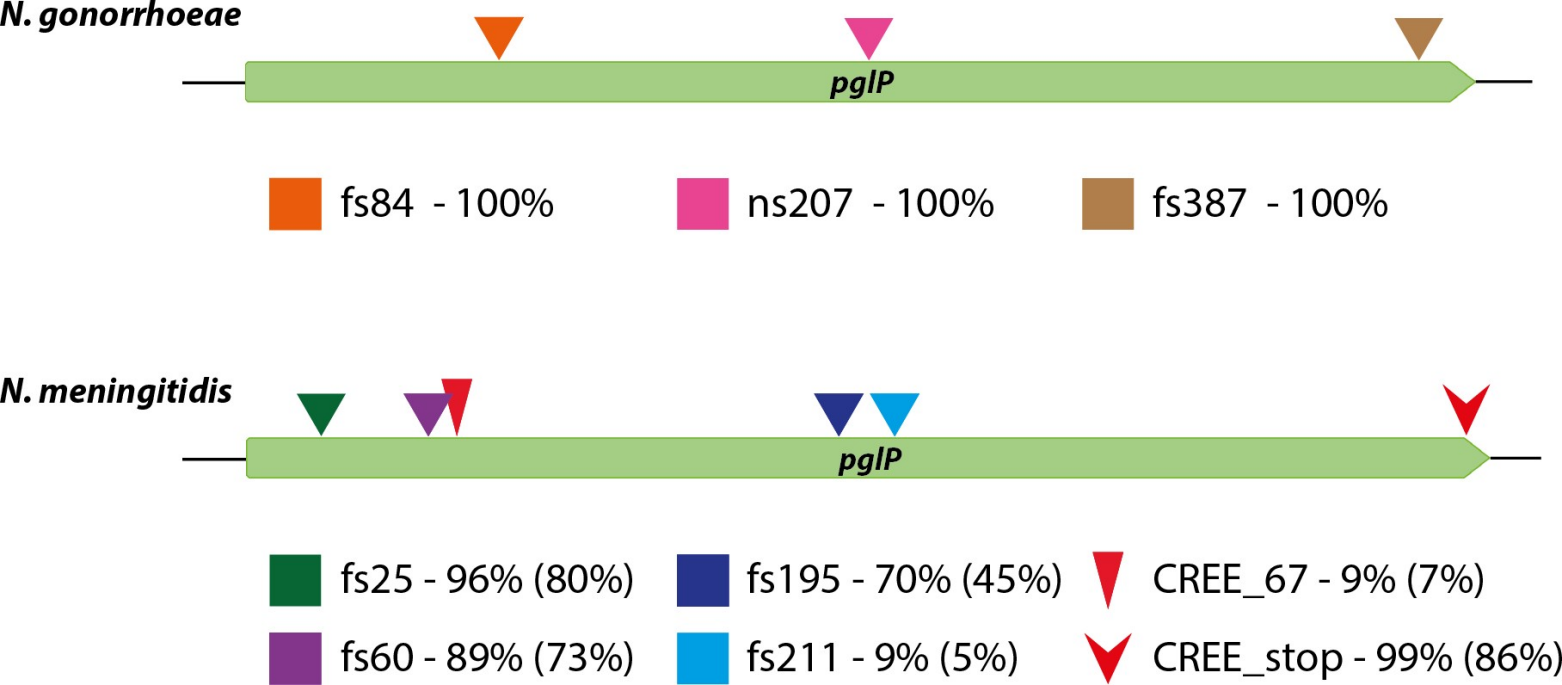
Pseudogenization of the *pglP* glycosyltransferase gene. Positions of ORF-disrupting SNVs in *N*. *gonorrhoeae* (top) and of ORF-disrupting SNVs and CREE insertions in *N*. *meningitidis* (bottom). For *N*. *gonorrhoeae*, the results include those from S1 Dataset as well an additional 833 genomes from gonococcal isolates of diverse geographic and temporal origins (S2 Dataset). For *N*. *meningitidis*, the percentage strains carrying particular disruptions are from strains in the 107 isolate strain collection representing global diversity in the latter half of the 20th century (Neisseria PubMLST database) followed in parentheses by those in 3567 isolates in the Meningitis Research Foundation Meningococcus Genome Library. Reductions in the percentage of ORF-disrupting mutations in the latter collection are associated with an over-representation of CC269 strains.

In stark contrast, all *pglP* alleles in *N*. *gonorrhoeae* and *N*. *meningitidis* contained ORF-disrupting mutations (Fig 6). *N*. *gonorrhoeae* strains shared a highly conserved allele containing three single nucleotide variants (SNV) generating chain-terminating mutations precluding PglP expression: frameshifts within codons 84 and 387 and a single base substitution creating a nonsense mutation at codon 207 (Fig 6, top panel). These results were confirmed using an additional 833 genomes from gonococcal isolates of diverse geographic and temporal origins (S2 Dataset). Among the set of 107 *N*. *meningitidis* genomes representative of disease-causing isolates from the latter half of the 20^th^ century, all but one *pglP* allele carried a Correia repeat enclosed element (CREE) inserted into the corresponding stop codon (Fig 6, bottom panel). CREEs are short, inverted-repeat containing, transposon-like elements distributed at high copy number within the genomes of all *N*. *gonorrhoeae* and N. *meningitidis* strains [43]. Together with the creation of an AT dinucleotide repeat associated with its insertion, the CREE results in the *pglP* ORF being extended by 18 amino acid residues. Furthermore, all but two alleles contained pseudogenizing SNVs resulting in ORF disruptions. These included those leading to frameshift mutations at codons 25, 60, 195 and 211 as well as alleles containing an additional CREE insertion between codons 67 and 68 (Fig 6, bottom). Further examination revealed that the vast majority of the *N*. *meningitidis* allele population encompassed various admixtures of the ORF-disrupting mutations, results that only can be accounted for by HGT and intragenic recombination (S3 and S4 Datasets). Assuming the presence of an intact allele reflects the ancestral state, the *pglP* pseudogenes and *pglP* absence are derived. In addition, isolates lacking *pglP* likely arose from a seminal deletion event that was subsequently disseminated by HGT across species (S5 Fig).

### Acquisition of an intact *pglP* allele in *N*. *meningitidis* CC269 isolates from commensal species

**Fig 7:**
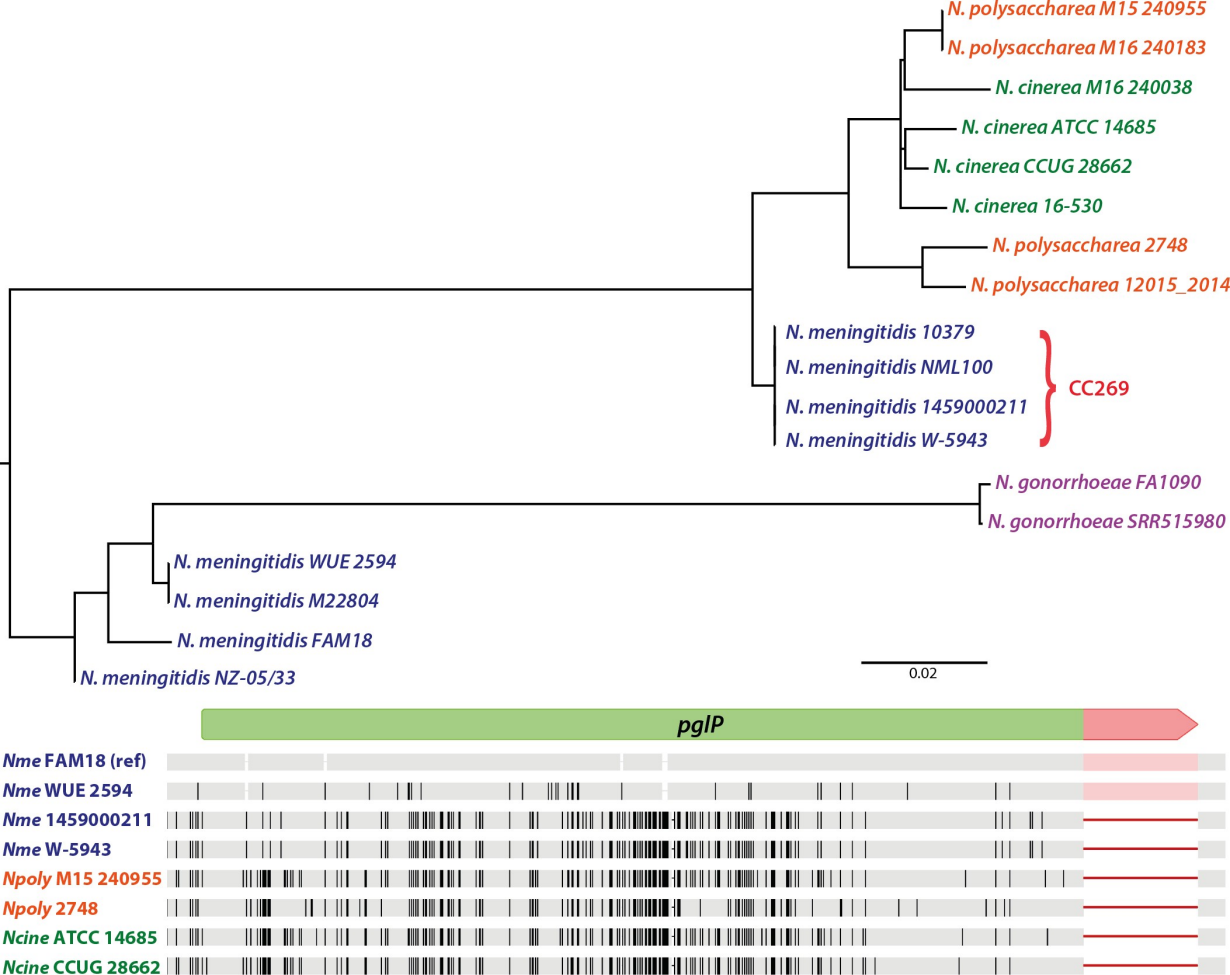
HGT-mediated acquisition of an intact *pglP* allele in CC269 strains. ORF nucleotide sequences from *pglP* alleles (excluding the ORF—extending CREE insertion) were aligned for phylogenetic sequence analyses using Clustal W (top panel). Multiple-alignment view of the variant nucleotide sites detected in *pglP* with that of FAM18 set as a reference sequence (bottom panel). The FAM18 allele contains the *C*-terminal, ORF—extending CREE insertion (segment in pink). Each SNP that differed from FAM18 is shown as a single line with thicker lines indicative of multiple neighboring SNPs. The sequence identities of another *N*. *meningitidis pglP* pseudogene, two CC269 alleles, two *N*. *polysaccharea* alleles and two *N*. *cinerea* alleles generated using ParSNP are shown. Note the absence of the CREE sequences in the CC269, *N*. *polysaccharea* and *N*. *cinerea* alleles and that greater than 80% of all *pglP* alleles in CC269 strains are 100% identical to those identified here.

The two *pglP* alleles lacking ORF-disrupting SNVs and the internal ORF-disrupting CREE found in the 107 *N*. *meningitidis* strain collection genomes were from clonal complex 269 (CC269) isolates. These two were 100% identical to one another at the nucleotide level and exhibited a distinct pattern of reduced similarity to the other *N*. *meningitidis* alleles. We analyzed a larger assemblage of 3567 isolates in which CC269 complex strains genomes were well represented and found over 81% of CC269 genomes carried identical intact *pglP* alleles (S4 Dataset). BLAST analyses using these alleles revealed higher identities to those from *N*. *polysaccharea* and *N*. *cinerea* strains than to other *N*. *meningitidis* strains. These affinities were confirmed by phylogenetic analysis revealing that CC269 *pglP* alleles clustered with the *N*. *polysaccharea* and *N*. *cinerea* alleles (Fig 7, top panel, S7 Fig). These findings were in clear contrast to the species—defined associations generated by core gene-based examination of the same strains (S8 Fig). The shared identities and a likely common source of these CC269 alleles were further confirmed by SNP density plot analysis revealing their nearly identical signatures (Fig 7, bottom panel). Together, the findings indicate that the intact CC269 *pglP* alleles arose by HGT involving a *N*. *polysaccharea* / *N*. *cinerea* donor source. Moreover, based on the SNP density analyses of the two flanking genes, the junctions of the DNA integration of this presumed one—time event extend outside the locus defined by the three genes (S9 Fig).

## Discussion

We used comparative genomics to reconstruct the evolutionary histories of protein glycosylation glycan biosynthesis in *N*. *gonorrhoeae* and *N*. *meningitidis*. The results show that current glycan repertoires in these species and congeners *N*. *polysaccharea* and *N*. *lactamica* result from acquisition of new glycosyltransferase genes coincident with loss of gene components of a progenitor pathway. We previously identified a conflict related to potential redundancy and competition for shared pathway intermediates by the PglA and PglH glycosyltransferases [15]. Together with evidence for hypomorphic *pglA* and *pglH* alleles, this led to the hypothesis that a conserved deletion inactivating both *pglG* and *pglH* in strains of *N*. *gonorrhoeae* and *N*. *meningitidis*, represented a resolution of this functional redundancy with the consequence of reduced glycan diversity [5, 15, 24]. In retrospect, it is clear that those polymorphisms are a mere subset of a larger number of genetic events associated with epistasis–involved gene decay, gene loss and ultimately replacement of a pre-existing glycan biosynthesis pathway.

Particularly striking in this context is the seeming continuum of inferred gene loss, pseudogenization and gene acquisition spanning the genus *Neisseria* in a pattern paralleling species group phylogenetic relationships. Here, critical delineating genetic events appear to be the loss of *pglJ* (encoding the dehydrogenase that generates the substrate UDP-GlcNAc from UDP-GlcNAcA) and the acquisition of *pglA* and *pglE* (encoding UDP-Gal utilizing glycosyltransferases) (Figs 2 and 3). Based on earlier findings, the absence of *pglJ* would be epistatic to both *pglG* encoding the glycosyltransferase utilizing UDP-GlcNAcA and *pglP* that targets the Und-PP-oligosaccharide terminating in GlcNAcA (generated by PglG) (Fig 2). Relaxed selection mediated by negative epistasis most likely accounts for the accumulation of ORF-disrupting SNVs and insertion elements in the *pglP* genes of *N*. *gonorrhoeae* and *N*. *meningitidis*. It is remarkable given the propensity for HGT between *N*. *gonorrhoeae* and *N*. *meningitidis* that none of the *pglP* mutations are shared between strains of these two species. This finding of parallel but independent evolutionary processes in *N*. *gonorrhoeae* and *N*. *meningitidis* is to our knowledge unprecedented and furthermore, indicates that *pglP* pseudogenization arose after the divergence of these species from a common ancestor. Although similar relaxed selection should be active on *pglG* in backgrounds lacking *pglJ*, none of the *N*. *gonorrhoeae* and *N*. *meningitidis* alleles have ORF-disrupting mutations. It is possible that such *pglG* alleles might accumulate missense mutations that preclude function but these are more difficult to infer from genomic sequence data alone. A further complicating factor is that the majority of *N*. *gonorrhoeae* and *N*. *meningitidis pglG* alleles are subject to high-frequency, on-off expression mediated by hypermutable, homopolymeric polyG repeat tracts. The ability to maintain *pglG* alleles in an off (out-of-frame) but reversible configuration might buffer against classical gene decay processes.

We suggest two non-exclusive scenarios to account for replacement of the ancestral *pglG/H/P* pathway by the *pglA/E* pathway. One would be that the presence of di- and trisaccharides terminating in galactose residues defined by the latter pathway might have altered function with regard to recognition by components of the innate or adaptive immune system or other glycan-associated phenotypes. This shift in function model might also relate to differences in the glycoprotein repertoires manifest in different species. For example, the most abundant glycoproteins in *N*. *gonorrhoeae* and *N*. *meningitidis* are the pilin subunits of their surface-displayed type IV pilus colonization factors while pilin is not subject to glycosylation in the deeply branching species *N*. *elongata* subsp. *glycolytica* group [25]. Another model would be that the alternate pathways come with variable metabolic costs where differences in the pools of UDP-sugars and/or Und-PP-linked saccharides exert pleiotropic effects on overlapping or converging pathways involving cell wall or LPS biosynthesis. We also examined the distribution of the *galE* gene encoding UDP-glucose 4-epimerase that carries out the reversible epimerization of UDP-glucose to UDP-galactose. *N*. *gonorrhoeae* and *N*. *meningitidis* (and likely other *Neisseria* species) cannot utilize exogenous sources of galactose, and GalE is required to generate the cognate substrates for PglA and PglE galactosyltransferases [44]. Intact *galE* alleles were found in all genomes of species carrying *pglA* and *pglE* but were also present in those of *N*. *cinerea* and *N*. *elongata* species groups isolates (S1 Dataset). They were differentially distributed in the *N*. *subflava* species groups isolates and absent from *N*. *mucosa* species group sequences (S1 Dataset). Thus, although *galE* is necessary for Gal-containing glycoforms, there is no strict correlation between its presence and glycoform status.

Along with work showing that some gonococcal strains can reacquire intact *porA* alleles from *N*. *meningitidis* [45], the results here confirm that intraspecies recombination can have significant consequences for pseudogene structure, distribution and stability. Moreover, the distribution of *N*. *meningitidis pglP* alleles bearing anywhere from one to five ORF-disrupting mutations is undoubtedly due to intragenic recombination. It is then impossible to determine the temporal order with which the mutations occurred or to infer the relative age of these pseudogenes by virtue of the number of accumulated mutations [46].

The widespread distribution of inactive or missing *pglP* alleles in *N*. *gonorrhoeae*, *N*. *meningitidis* and *N*. *lactamica* isolates suggest that as clades, the species groups may have undergone relatively recent reductions in population size (with the altered allele-bearing strain being first to pass the bottleneck). There may have been a selective sweep of the defective or missing allele through the population via natural genetic transformation. While it is difficult to differentiate between these possibilities, it is worthwhile noting that *pglP* status and allele distribution might be impacted by factors unrelated to PglP function per se [46]. This consideration may especially apply to the situation in *N*. *meningitidis* where the most prevalent gene disrupting mutation is the CREE insertion within the stop codon. CREEs contain active promoter elements [43] and hence, their presence there could alter transcription of the downstream gene that in this case is *phoH*. In fact, RNA-SEQ analyses reported that the *phoH* transcription start site (TSS) in such a strain occurs within the CREE [47]. It remains unclear what function PhoH serves and if the *C*-terminal ORF extension resulting from the CREE insertion perturbs PglP activity. Nonetheless, it is plausible that non-neutral forces may be driving the distribution and retention of the *pglP* pseudogene in *N*. *meningitidis*. Although the TSS for wildtype *phoH* has yet to be determined for any *Neisseria* species, *pglP* deletion might likewise impact *phoH* expression.

The results here also emphasize the potential for HGT to generate unbalanced or discordant polymorphisms due to the multi-locus nature of *pgl* gene networks. These findings contrast strongly with the genetic events underlying capsule serotype/serogroup switching in *Streptococcus pneumoniae* and *N*. *meningitidis* that involve recombination events spanning a single, large locus in a “plug and play”-type switching process [2, 48]. Discordant gene interactions are particularly evident in the case of *N*. *polysaccharea* isolates where some strains carry seemingly incompatible gene sets such as intact *pglP* alleles in backgrounds lacking *pglG/H* and/or *pglJ*. In fact, despite the limited number of genomes examined, *N*. *polysaccharea* strains exhibit extreme levels of diversity in *pgl* gene status that appear to result from interspecies HGT. These findings are consonant with others showing that *N*. *polysaccharea* isolates form a polyphyletic group [9, 25]. It is also striking that the *pgl* gene content of isolates within this single species group encompasses the majority of patterns seen at the macroevolutionary level. Together with the other findings here, we suggest that *N*. *polysaccharea* may act as a nexus for gene flow bridging pathogen and commensal species.

Another example of discordant *pgl* polymorphisms generated by recombination is found in the CC269 strains possessing an intact *pglP* allele. These strains all carry the *pglG/H* deletion polymorphism and lack *pglJ*, conditions that would preclude PglP function (S2 and S3 Datasets). Thus, if there is a selective advantage imparted by the genome import event, it likely relates to a linked gene with which *pglP* hitchhikes. Given this situation, the current prevalence of CC269 strains as causes of *N*. *meningitidis* invasive disease [49] and the seemingly strong selective pressure for *pglP* gene decay evident in other *N*. *meningitidis* strains, it will be of interest to assess the fate of *pglP* in CC269 lineage isolates over time.

## Materials and methods

### Bacterial strains and culture conditions

*N*. *elongata* subsp. *glycolytica* ATCC 29315 was used for mutagenesis and genetic complementation studies involving *pglP* [62]. Other bacterial strains used in this study are described in S1 Table and were grown on conventional GC medium as described previously [50]. Antibiotics were used for selection of *Neisseria elongata* subsp. *glycolytica* transformants at the following concentrations: streptomycin, 750 μg/mL; kanamycin, 50 μg/mL; and chloramphenicol, 10 μg/mL.

### Directed mutagenesis of *Nelon_11110* and *Nelon_11105* in *N*. *elongata* subsp. *glycolytica*

The region encompassing *Nelon_11110* and flanking sequences from strain KS944 (*N*. *elongata* subsp. *glycolytica* ATCC 29315) was PCR amplified using primers av2934 and av2935 and the resulting product TA-cloned into the pCR2.1-TOPO vector. DNA of the resulting plasmid pAK220 was digested with HincII and StuI (to delete a 587bp intragenic region of the *Nelon_11110* ORF) and ligated with the *kanR* gene cassette from pKan (generated by HincII digestion) to generate AK227. A similar strategy was used to disrupt *Nelon_11105* where the *kanR* cassette was inserted 325bp into the ORF while concurrently deleting the ORF *C*-terminus to generate strain NW270. Flanking regions to *Nelon_11105* were PCR amplified using primers nw180/nw181 and nw184/nw185 and Gibson assembled to the *kanR* cassette amplified with primers nw188/nw189. These constructs (S1 Fig) were introduced by transformation into *N*. *elongata* subsp. *glycolytica* strain KS992 (that carries a *nirK*-*His* allele (in which the NirK ORF is translationally fused to a 6Xhistidine *C*-terminal extension). The strains, plasmids and oligonucleotide primers used here are found in S1 and S2 Tables.

### Allelic exchange of the *pglP* locus in *N*. *elongata* subsp. *glycolytica*

The introduction of defined, marker-less *pglP* alleles into *N*. *elongata* subsp. *glycolytica* was performed through modification of a previously established two-step mutagenesis strategy (S1 Fig). The method uses a two-gene cassette containing both a selectable marker and a counter selectable marker (*rpsL*^*+*^). The gene cassette originally employed in *N*. *gonorrhoeae* utilizes an *ermC*´ as a selectable marker [51]. As selection for the erythromycin resistance marker in *N*. *elongata* subsp. *glycolytica* was problematic, a modified gene cassette was constructed in which the *ermC*´gene was replaced by a *kanR* gene cassette. This was done by first digesting pFLOB4300 with SacI and NsiI (to release *ermC’*) and enzymatic treatment to generate blunt ends. This fragment was then ligated to the HincII digestion-generated fragment containing *kanR* from pKan to generate plasmid pKP79. To generate the streptomycin resistance background in *N*. *elongata* subsp. *glycolytica*, strain KS944 was transformed with *rps* marker DNA from *N*. *gonorrhoeae* strain N400 (that naturally carries the streptomycin resistance point mutation changing amino acid 43 of 30S ribosomal protein S12 from a lysine to an arginine). The ensuing strain (NK2259) was then transformed so as to carry the *nirK*-*His* allele from strain KS992 to generate strain NW37. Transformation of NW37 with pKP79 DNA and selection for kanamycin resistance (generating strain NW154) resulted in the replacement of the *pglP* ORF by the *kanR*/*rpsL*^*+*^ gene cassette and concurrent streptomycin sensitivity. Transformation of NW154 with DNA bearing homologous sequences flanking *pglP* and selection for streptomycin resistance results in precise allelic replacement of the *pglP* ORF. Donor DNAs used for allelic replacement were generated by PCR and Gibson assembly. To generate strain NW180 carrying an in–frame deletion encompassing residues 74–148 of the *pglP* ORF, primer pairs nw92/nw153 and nw154/nw98 were used to generate overlapping PCR products and Gibson assembled. To generate strain NW182 carrying the *pglP* allele ORF from *N*. *oralis* strain F0314, primer pairs nw92/111, and nw114/98 were used for PCR of the *pglP* flanking sequences from KS944 while primer pair nw112/113 was used to PCR the *N*. *oralis pglP* ORF (*HMPREF9016_01275*). These fragments were Gibson assembled and amplified by PCR. To generate strain NW212 carrying the *pglP* allele ORF from *N*. *cinerea* strain ATCC 14685, primer pairs nw92/152 and nw122/98 were used for PCR of the *pglP* flanking sequences from KS944 while primer pair nw151/121 was used to PCR amplify the *N*. *cinerea pglP* ORF (*NEICINOT_04976*). These fragments were Gibson assembled and amplified by PCR. As a positive control to “rescue” the wildtype allele, strain NW254 was generated by transformation using genomic DNA from strain KS944. All constructs were introduced into NW154 by transformation with selection for streptomycin resistance, scored for kanamycin sensitivity and verified by PCR and DNA sequencing. The strains, plasmids and oligonucleotide primers used here are found in S1 and S2 Tables.

### Genome analyses and bioinformatics

The presence and status of *pgl* genes within genomes from isolates across the genus were determined using BLASTn and BLASTp queries of genome sequences using the *Neisseria* PubMLST (http://pubmlst.org/neisseria/) and Meningitis Research Foundation Meningococcus Genome Library (http://www.meningitis.org/research/genome) databases. Forward searches utilized defined *pgl* alleles from *N*. *gonorrhoeae* and *N*. *elongata* subsp. *subspecies glycolytica* (S3 Table). Specific genomes / strains utilized are found in S1–S4 Datasets. To identify potential distant orthologues, the BLAST *E* score cutoff was set to 10^−5^ and sequence alignments were manually examined. Microsynteny at discrete loci was assessed by monitoring Blast hits with nucleotide sequence start and end coordinates within defined sequence bins. Microsynteny was further validated using the compare region viewer function in PATRIC [52], the gene cluster function in KEGG gene database [53] and where necessary, local genome alignment using progressiveMauve [54]. For assessing the relatedness of *pglP* alleles and associated intraspecies HGT, a reference nucleotide sequence from FAM18 (AM421808.1) comprising the region from the periplasmic protein (NMC0788) to *phoH* (NMC0784) was used in a BLASTn query with default parameters against a BLAST database built using the contigs from selected PubMLST *Neisseria* genomes. Top hits were extracted from the contigs. Nucleotide sequences were aligned using MAFFT (version 7.017) [55] and a phylogenetic tree of PglP constructed in Geneious 9.1.7 (https://www.geneious.com) using the RAxML plugin [56]. The phylogeny was visualized and annotated in FigTree (http://tree.bio.ed.ac.uk/software/figtree/). The SNP density plot was generated in and exported from Geneious. Further details of *pglP* phylogenetic analyses including SNV distribution determination are found in S3 and S4 Datasets.

### Analyses of Single Nucleotide Variant (SNV) and CREE distribution in gonococcal and meningococcal *pglP* Alleles

The distribution of specific ORF-disrupting polymorphisms was determined by BLASTN and BLASTP analyses using a control set of *pglP* gene and ORF sequences with default settings in BIGSdb / Neisseria PubMLST. In conjunction with these methods, focused BlastN searches using SNV-specific oligonucleotide sequences were employed (S1 Text). 100% scores indicated presence of a SNV or CREE presence. Isolates with poorer hits (90–99%) were manually checked for absence or presence of a mutation. Data for specific strains can be found in S3 and S4 Datasets.

### SDS/PAGE, immunoblotting and affinity purification of NirK

Procedures for protein electrophoresis, immunoblotting and purification of NirK-His-tagged proteins have been previously described [26].

### Targeted mass spectrometric glycan analyses

Conditions for the MS-based analyses of glycosylated NirK using in-gel protein, reverse-phase liquid chromatography- tandem MS (LC-MS2) analysis of proteolytic peptides, electron transfer dissociation (ETD) experiments and data analyses have been previously defined [26].

### LC-MS analysis of protein glycans using membrane extracts from commensal strains

Periplasmic and cytosolic protein fractions were generated as previously described [29] with the following modifications: protein precipitates were washed 10 times with 50mM pH 7.8 TEAB buffer (buffer B) utilizing a 3K cut-of Amicon prior to enzymatic digest. Protein concentration was determined twice on a Qubit and adjusted to 400μg for all protein samples. Adjusted protein samples were resuspended, reduced (DTT 10mM) for 30 min and alkylated (IAA 20mM) for 30 min (dark) in buffer A (buffer B + 6M urea, 1.5M ThioUrea with proteinase Inhibitor (PI, Roche complete EDTA free) and Phosphatase inhibitor (PhosI, Roche phosphostop EasyPack) in 50mM sodium orthovanadate) on a 10KDa cut-of spin filter (Amicon). Reduced and alkylated proteins were washed with Buffer B by spin filtering. Subsequent digest was done in 200μl buffer B, adding 2U of LysC (RT for 2H) before overnight digestion with 3% w/w trypsin at 37°C. Digested peptide samples were moved to a new low bind Eppendorf tube, added 2% FA and spun at 14K g for 10 min to precipitate lipids. The supernatants were transferred to a new tube for TiO2 (titanium dioxide) and SIMAC (sequential elution from Immobilized metal affinity chromatography) purification. TiO2 and SIMAC affinity purification were essentially done as previously described in reverse order. i.e the final TiO2 (high pH eluate was used for the SIMAC affinity purification, leaving a total of 4 samples. Each sample were desalted with R2/R3 as described [57]. Each sample was lyophilized prior to analysis on the Thermo Orbitrap Fusion. The dried peptides were dissolved in 0.1% formic acid and injected into an in-house packed 17 cm × 100 μ m Reprosil-Pur C18-AQ column (3 μ m; Dr. Maisch GmbH, Germany) using an Easy-LC nano-HPLC (Thermo Scientific,Germany).

Further details of MS-based characterization of glycoprotein-derived glycans are available upon request.

## Supporting information

S1 Fig Constructs and strategies used for mutagenesis and manipulation of the *pglP* gene/locus status in *N. elongata* subsp. *glycolytica* ATCC 29315 (KS944). (A, B). Detection of the NirK-His glycoprotein in *N*. *elongata* subsp. *glycolytica pgl* mutant / variant backgrounds by immunoblotting with polyHis-epitope recognizing mAb (C). WT: KS944; *pglC*: KS994, *pglPS74-R148*: NW180; *pglP*::*kan/rpsL+*: NW154; *pglP* rescue: NW254; *pglP*_*N*. *oralis*_: NW182 and *pglP*_*N*. *cinerea*_: NW212. Multiple isoforms of NirK-His are the result of macrohereogeneity (variable glycan site occupancy) as NirK has five sites of glycan occupancy.
(TIF)

S2 Fig Genomic organization of *pgl* genes in strains from *Neisseria* species groups. Genomes shown as lacking *pglG* and *pglH* retain the canonical *pglG3´*/*H5´* spanning deletion. The asterisks for *pglP* denote a pseudogene. Other genes are annotated as follows: diagonal line fill = O-antigen ligase like (UNIPROT D7N379 in *N*. *oralis*); horizontal line fill = HAD hydrolase (UNIPROT D7N385 in *N*. *oralis*); vertical line fill = formyl transferase (UNIPROT D7N386 in *N*. *oralis*) and blank fill = three unannotated ORFs (NELON_10550, NELON_10555 and NELON_10560 in *N*. *elongata* subspecies *glycolytica*).
(TIF)

S3 Fig Microsynteny at *pglA* loci in select species groups. % values are percentage of strains in the species group with that configuration. Other genes shown are annotated as encoding 3-oxoacyl-[acyl-carrier-protein] synthase 2 (KASII in *Ngo—*UNIPROT Q5F603), a transposase (IS in *Nme and Npo—*IS110) and a potassium transporter (*kefC* in *Npo—*UNIPROT E2PBV4).
(TIF)

S4 Fig Microsynteny at *pglE* loci in select species groups. % values are percentage of strains in the species group with that configuration. Other genes shown are annotated as encoding a putrescine-binding periplasmic protein (*potF* in *Ngo—*UNIPROT Q5FA28) and an uncharacterized protein (blue in *Nlact—*UNIPROT E4ZEM6)
(TIF)

S5 Fig Microsynteny at *pglP* loci in select species groups. % values are percentage of strains in the species group with that configuration. Other genes shown are annotated as encoding an uncharacterized protein (grey in *Ngo–*UNIPROT Q5F9H4), a metalloprotease (*pmbA* in *Nsub -*UNIPROT C0EPK5), an NADH-dependent flavin oxidoreductase (*nox* in *Nmuc–*UNIPROT F9EUJ4) and an uncharacterized protein (brown in *Nmuc*—UNIPROT F9EUJ7).
(TIF)

S6 Fig Alignment of representative PglP alleles/ORFs. Selected PglP alleles/ORFs were aligned with MAFFT using Geneious and subsequently used to generate an identity and a similarity—based matrix. The two tables were imported into Excel and combined into a single panel (top). Alleles of *pglP* were selected from across the genus as representatives of each species group, aligned using MAFFT and visualized using Jalview (bottom). Strains in which the allele is located within the core *pgl* locus begin with an asterix (*) and protein regions with >90% conservation are highlighted in red. The locations of the two glycosyltransferase domains (as predicted by NCBI) are underlined in the alignment (blue line = pfam13439 and green line = pfam00534).
(TIF)

S7 Fig Phylogenetic analyses of *pglP* alleles. A maximum likelihood phylogenetic tree of aligned *pglP* nucleotide sequences was generated using MEGA (Molecular Evolutionary Genetics Analysis) V7 using the Tamura-Nei model [58]. A total of 500 bootstrap iterations were undertaken allowing a confidence interval for each node to be determined. The resulting consensus tree was then annotated for each *Neisseria* species. The analysis involved 204 nucleotide sequences.
(TIF)

S8 Fig Genus-level phylogeny of selected strains of relevance to *pglP* allele HGT and diversity using a core genome alignment. *De novo* assemblies from PubMLST were annotated using Prokka (version 1.14) [59]. A genus-level phylogeny was constructed with FastTree (version 2.1.11) [60] using a core genome alignment generated by Roary (version 3.12.0) [61] with a minimum BLASTP identity of 90%.
(TIF)

S9 Fig SNP analyses across *pglP* and flanking gene loci reveals the extent of interspecies import into *N. meningitidis* CC269 isolates. See Fig 7 and text for further details.
(TIF)

S1 Table Lists of strains and plasmids used in this study. (PDF)

S2 Table PCR oligonucleotide primers. (PDF)

S3 Table Pgl protein / gene sequences used in iterative BLASTP and BLASTN queries. (PDF)

S1 Text Oligonucleotide sequences used to assess *pglP* pseudogene mutation distribution. (PDF)

S1 Dataset Panel of strains and isolates from *Neisseria* species and species groups used in primary analyses of *pgl and galE* gene status. Gonococcal isolates used originate from those used in a prior study of protein antigen distribution across the genus [63]. Meningococcal isolates originate from a public database which represents global meningococcal diversity in the 20^th^ century. The remaining species include all respective isolates with complete genome sequences available at the *Neisseria* PubMLST database at the time of this work. Colored cells indicate presence of a gene, crossed cells (X) denote out-of-frame alleles (exclusive of those resulting from phase variable mutational hotspots, the presence of transposon elements is marked with TN, diagonal lines (/) indicate that gene is partially present and isolates with two alleles are marked with 2. Isolate ID numbers are derived from the *Neisseria* PubMLST database.
(XLSX)

S2 Dataset Panel of 833 diverse strains and isolates of *N. gonorrhoeae* whose genomes were used in analyses of *pglP* SNV distribution. Table shows strain name, date and country of isolation and multi-locus sequence type (MLST). Isolate ID numbers are derived from the *Neisseria* PubMLST database.
(XLSX)

S3 Dataset Panel of 107 strains and isolates of *N. meningitidis* representing global meningococcal diversity in the 20th century whose genomes are used in analyses of *pglP* allele distribution. Table shows strain name, date and country of isolation, serogroup, as well as sequence type and clonal complex designation. Colored cells indicate the presence of ORF–disrupting SNVs or CREE insertions (see Fig 6 for details). ID numbers are derived from the *Neisseria* PubMLST database.
(XLSX)

S4 Dataset Panel of 3567 strains and isolates of *N. meningitidis* whose genomes come from Meningitis Research Foundation Meningococcus Genome Library and used here in analyses of *pglP* allele distribution. Table shows strain name, date and country of isolation, serogroup, as well as sequence type and clonal complex designation. Colored cells indicate the presence of ORF–disrupting SNVs or CREE insertions (see Fig 6 for details). ID numbers are derived from the *Neisseria* PubMLST database.
(XLSX)
