# Supplementary figures and images for: Genetic determinants of genus-level glycan diversity in a bacterial protein glycosylation system
Source: PLoS Genet. 2019 Dec 23;15(12):e1008532. doi: 10.1371/journal.pgen.1008532 (PMC6959607; doi:10.1371/journal.pgen.1008532)

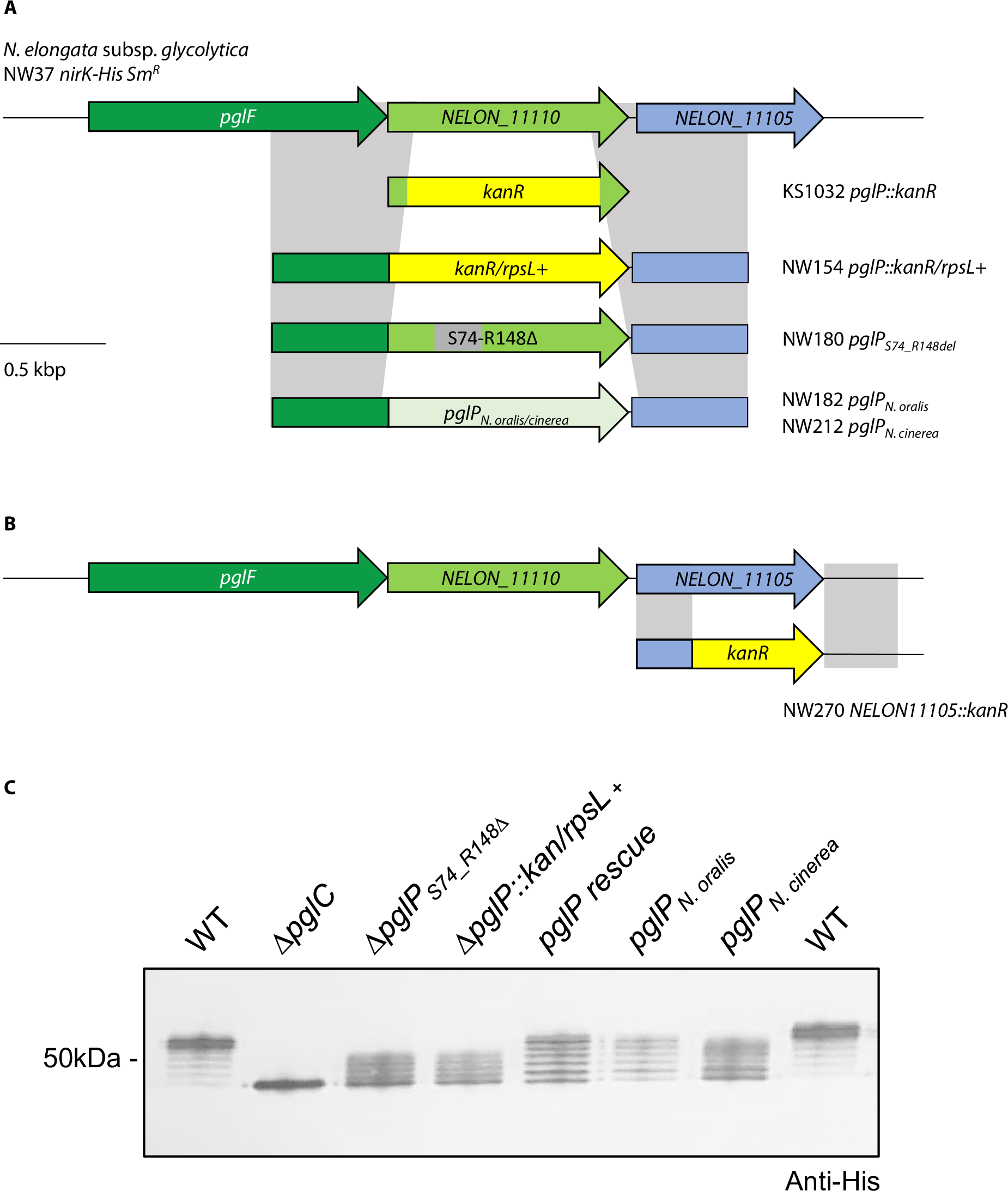

Supplement: S1 Fig — (A, B). Detection of the NirK-His glycoprotein in N. elongata subsp. glycolytica pgl mutant / variant backgrounds by immunoblotting with polyHis-epitope recognizing mAb (C). WT: KS944; pglC: KS994, pglPS74-R148: NW180; pglP::kan/rpsL+: NW154; pglP rescue: NW254; pglPN. oralis: NW182 and pglPN. cinerea: NW212. Multiple isoforms of NirK-His are the result of macrohereogeneity (variable glycan site occupancy) as NirK has five sites of glycan occupancy. (TIF) [file pgen.1008532.s001.tif]

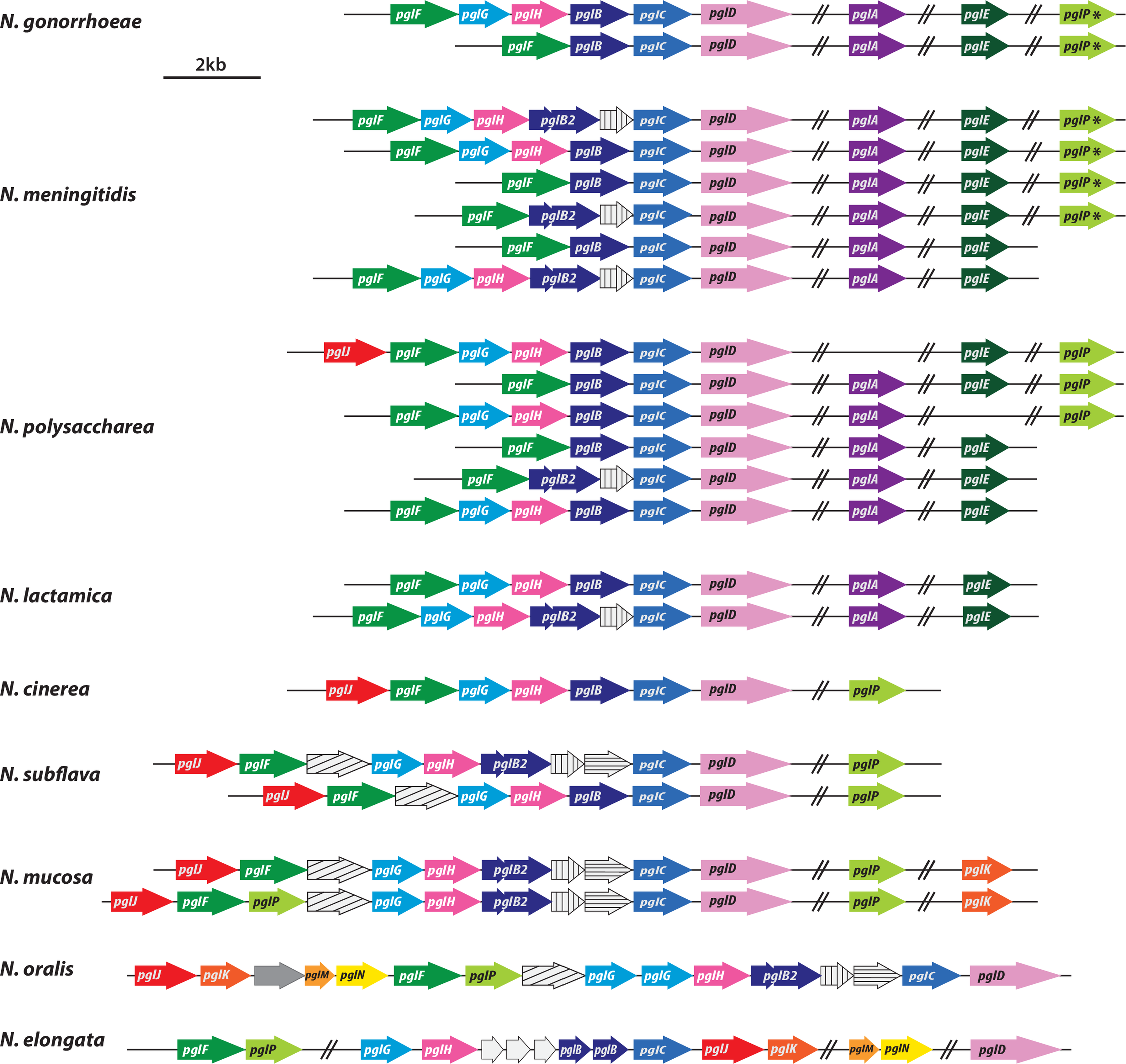

Supplement: S2 Fig — Genomes shown as lacking pglG and pglH retain the canonical pglG3´/H5´ spanning deletion. The asterisks for pglP denote a pseudogene. Other genes are annotated as follows: diagonal line fill = O-antigen ligase like (UNIPROT D7N379 in N. oralis); horizontal line fill = HAD hydrolase (UNIPROT D7N385 in N. oralis); vertical line fill = formyl transferase (UNIPROT D7N386 in N. oralis) and blank fill = three unannotated ORFs (NELON_10550, NELON_10555 and NELON_10560 in N. elongata subspecies glycolytica). (TIF) [file pgen.1008532.s002.tif]

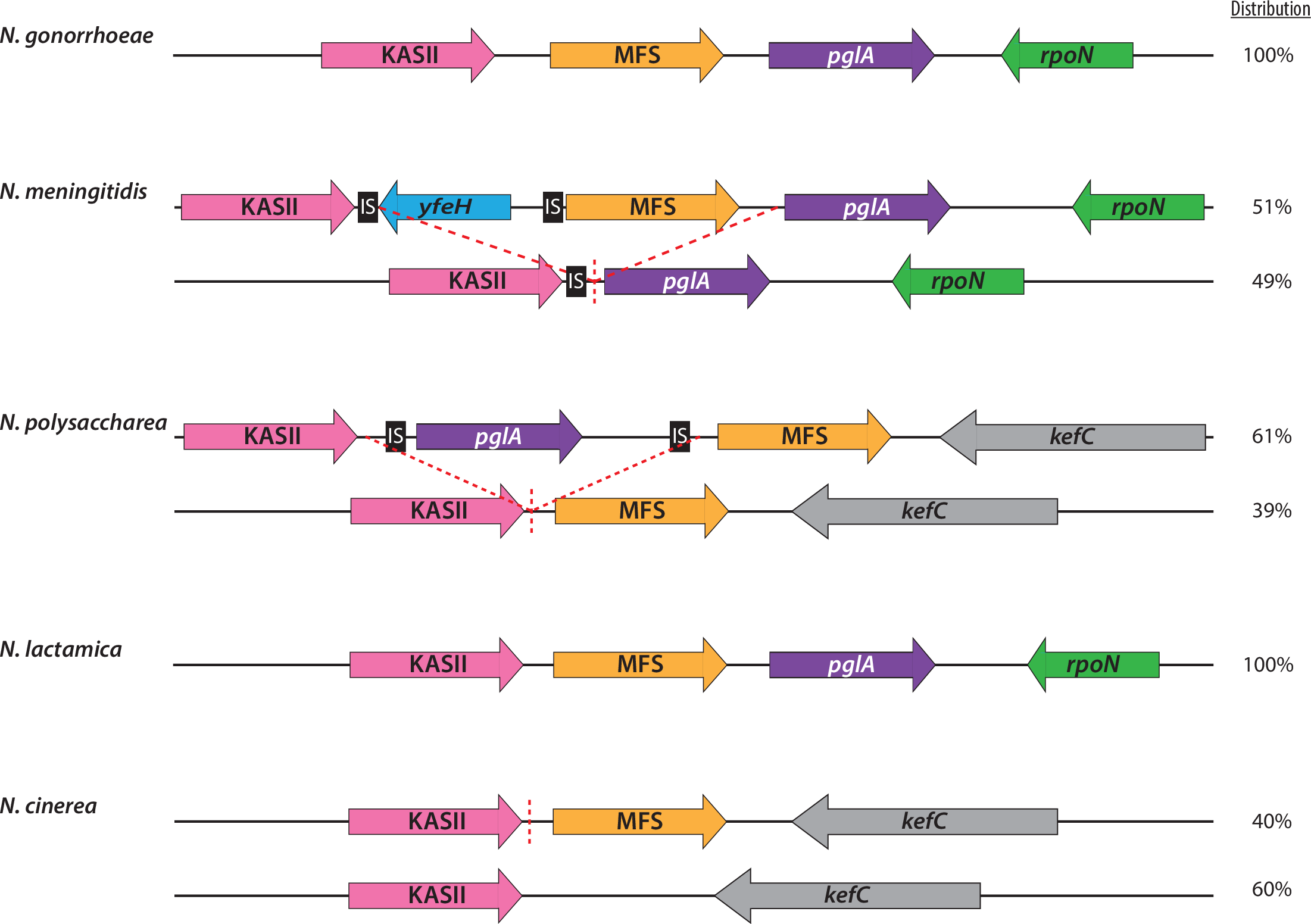

Supplement: S3 Fig — % values are percentage of strains in the species group with that configuration. Other genes shown are annotated as encoding 3-oxoacyl-[acyl-carrier-protein] synthase 2 (KASII in Ngo—UNIPROT Q5F603), a transposase (IS in Nme and Npo—IS110) and a potassium transporter (kefC in Npo—UNIPROT E2PBV4). (TIF) [file pgen.1008532.s003.tif]

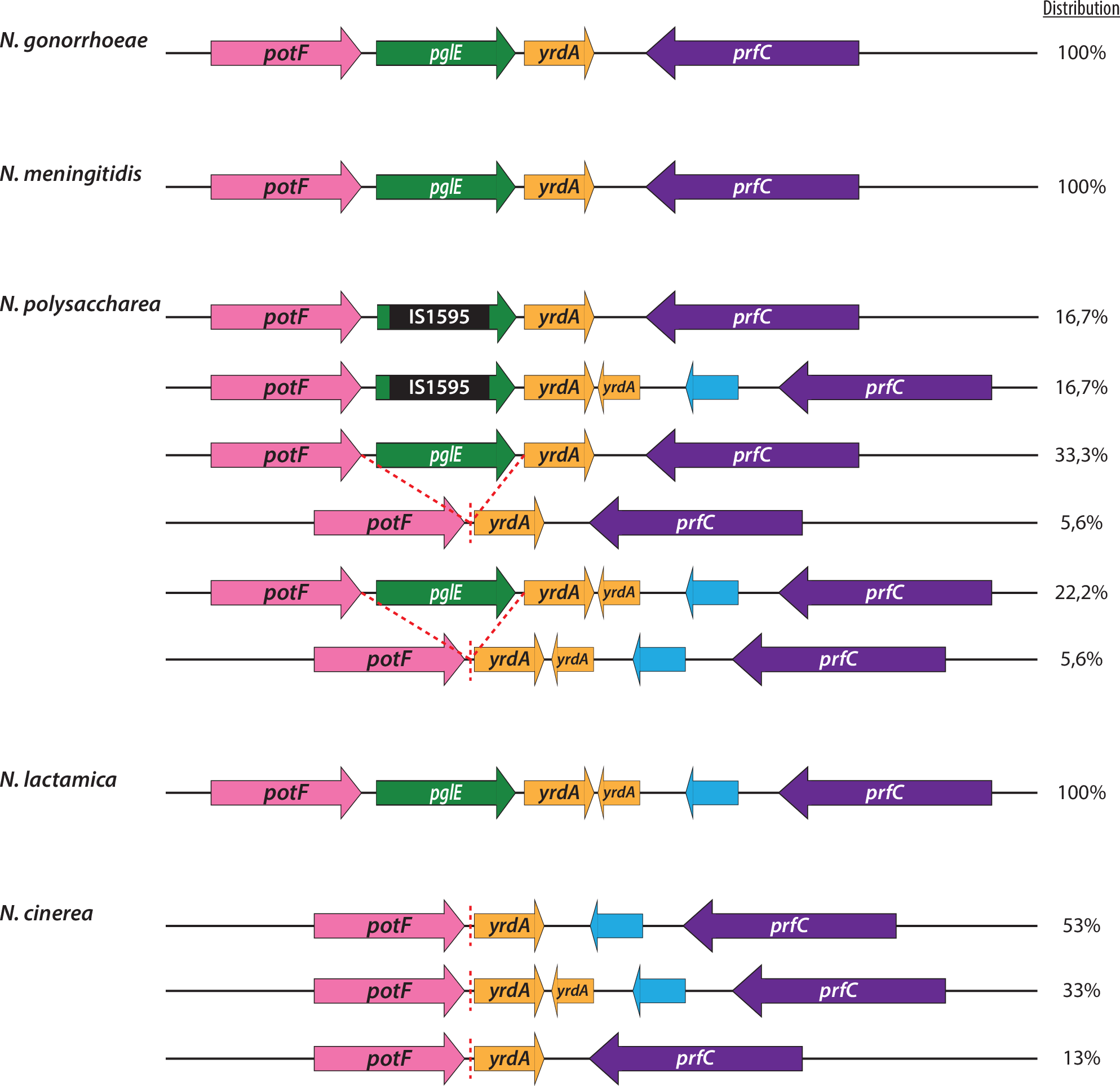

Supplement: S4 Fig — % values are percentage of strains in the species group with that configuration. Other genes shown are annotated as encoding a putrescine-binding periplasmic protein (potF in Ngo—UNIPROT Q5FA28) and an uncharacterized protein (blue in Nlact—UNIPROT E4ZEM6) (TIF) [file pgen.1008532.s004.tif]

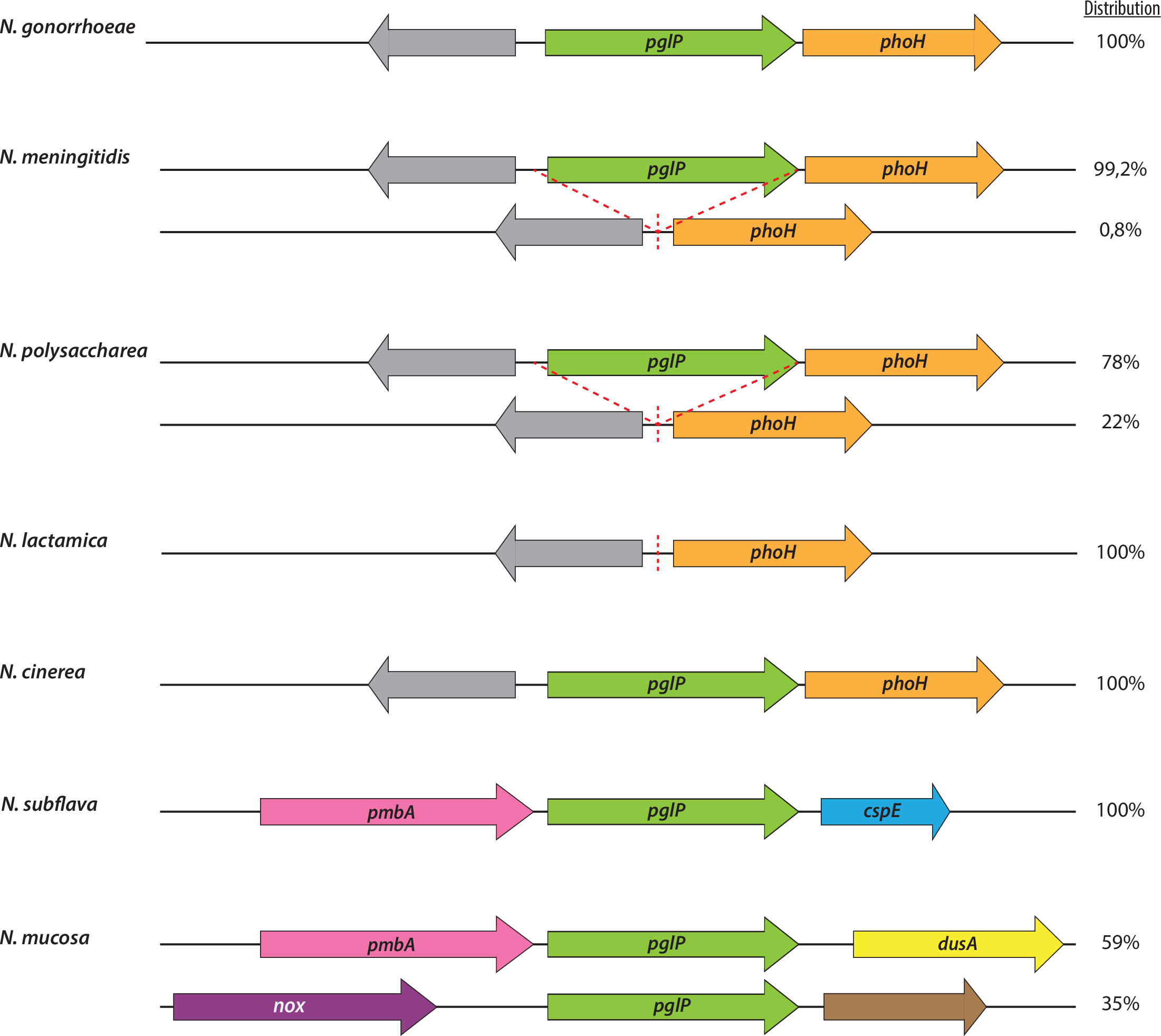

Supplement: S5 Fig — % values are percentage of strains in the species group with that configuration. Other genes shown are annotated as encoding an uncharacterized protein (grey in Ngo–UNIPROT Q5F9H4), a metalloprotease (pmbA in Nsub -UNIPROT C0EPK5), an NADH-dependent flavin oxidoreductase (nox in Nmuc–UNIPROT F9EUJ4) and an uncharacterized protein (brown in Nmuc—UNIPROT F9EUJ7). (TIF) [file pgen.1008532.s005.tif]

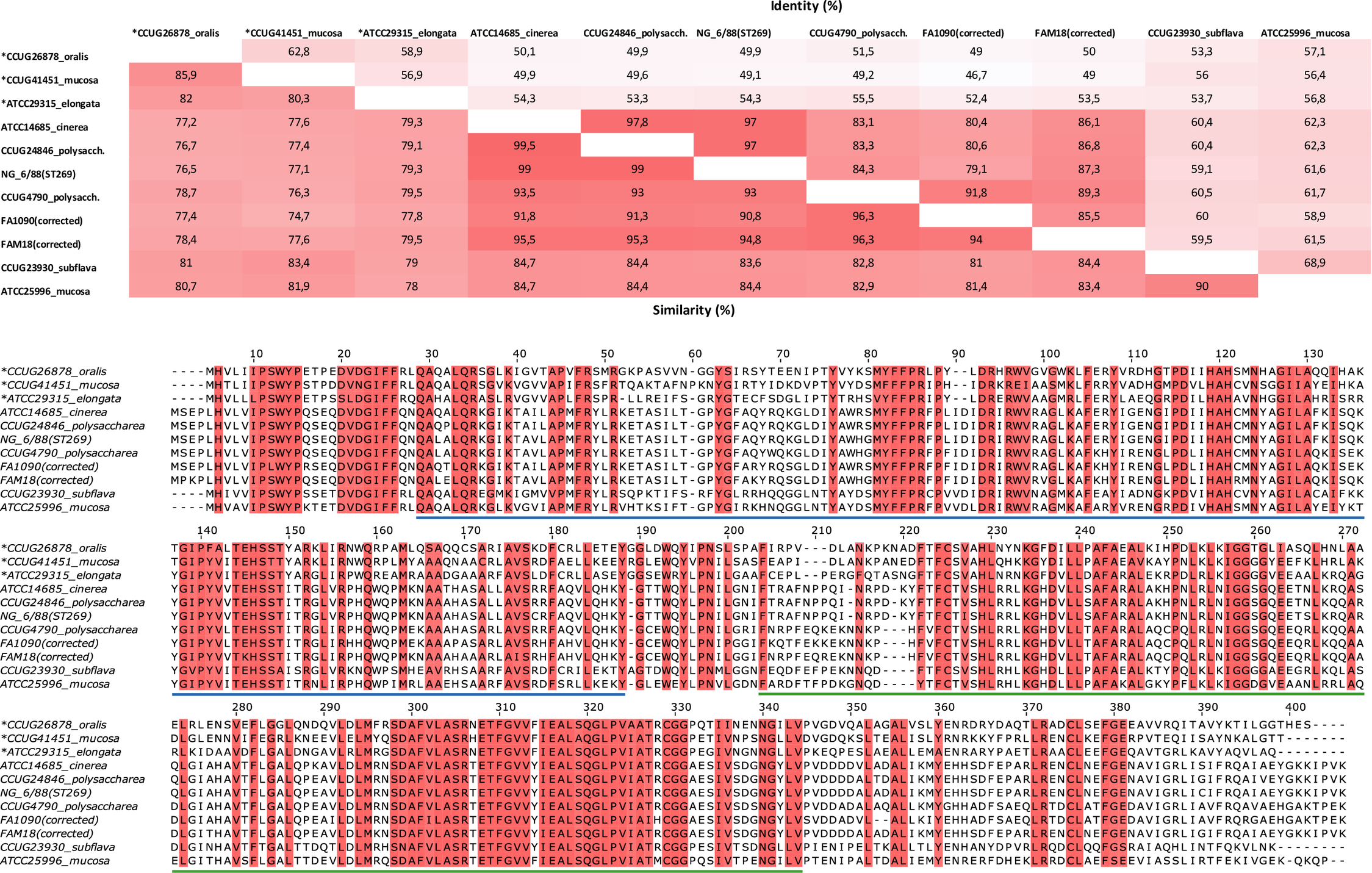

Supplement: S6 Fig — Selected PglP alleles/ORFs were aligned with MAFFT using Geneious and subsequently used to generate an identity and a similarity—based matrix. The two tables were imported into Excel and combined into a single panel (top). Alleles of pglP were selected from across the genus as representatives of each species group, aligned using MAFFT and visualized using Jalview (bottom). Strains in which the allele is located within the core pgl locus begin with an asterix (*) and protein regions with >90% conservation are highlighted in red. The locations of the two glycosyltransferase domains (as predicted by NCBI) are underlined in the alignment (blue line = pfam13439 and green line = pfam00534). (TIF) [file pgen.1008532.s006.tif]

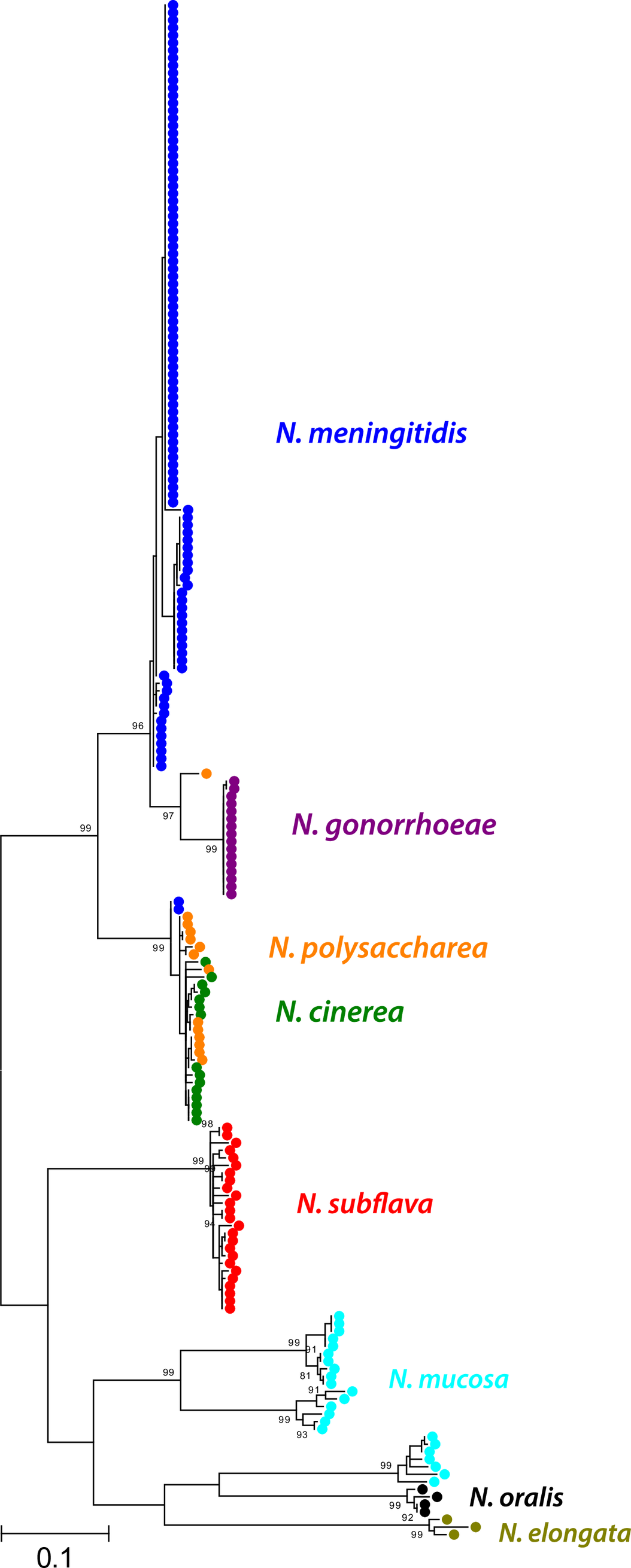

Supplement: S7 Fig — A maximum likelihood phylogenetic tree of aligned pglP nucleotide sequences was generated using MEGA (Molecular Evolutionary Genetics Analysis) V7 using the Tamura-Nei model [58]. A total of 500 bootstrap iterations were undertaken allowing a confidence interval for each node to be determined. The resulting consensus tree was then annotated for each Neisseria species. The analysis involved 204 nucleotide sequences. (TIF) [file pgen.1008532.s007.tif]

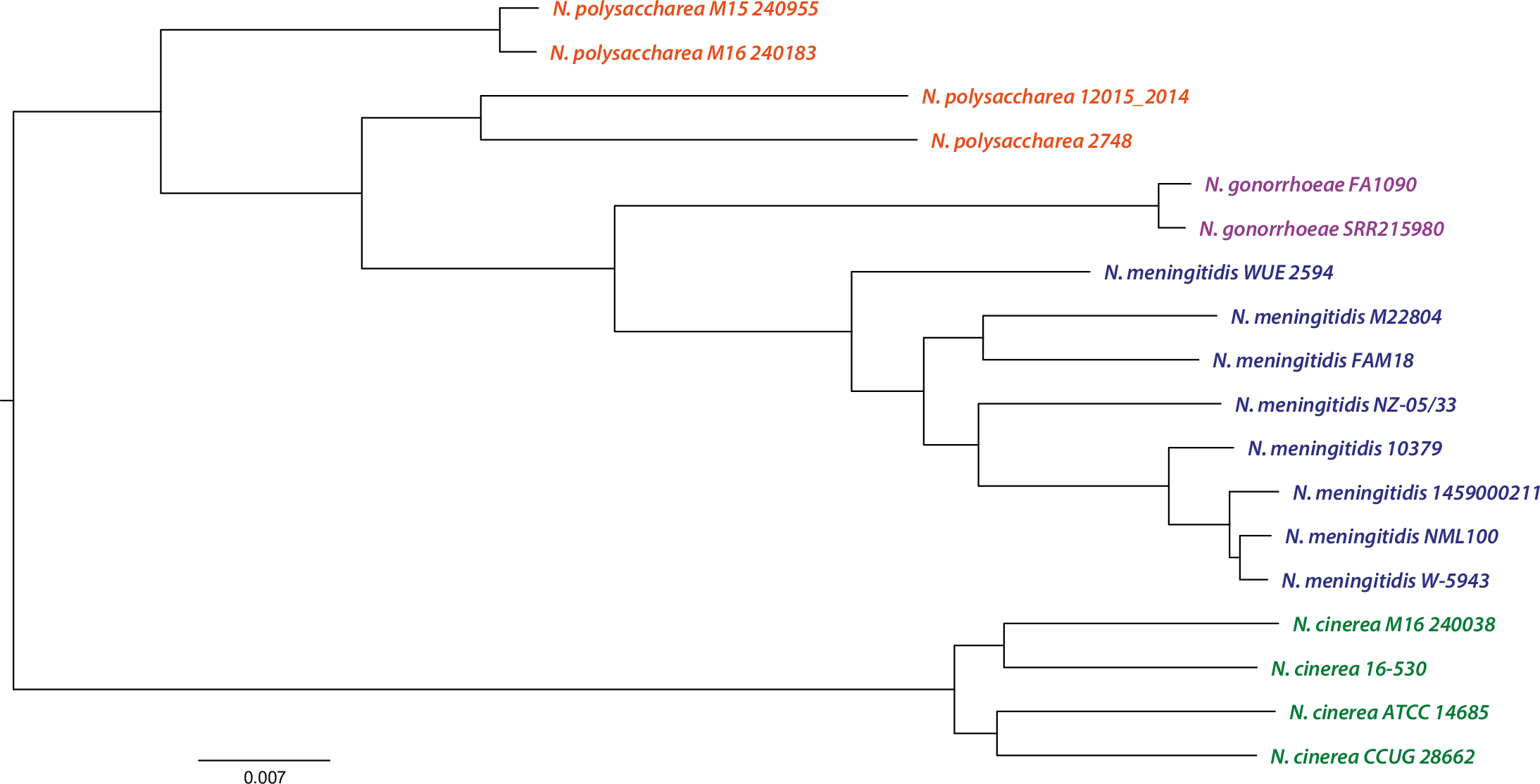

Supplement: S8 Fig — De novo assemblies from PubMLST were annotated using Prokka (version 1.14) [59]. A genus-level phylogeny was constructed with FastTree (version 2.1.11) [60] using a core genome alignment generated by Roary (version 3.12.0) [61] with a minimum BLASTP identity of 90%. (TIF) [file pgen.1008532.s008.tif]

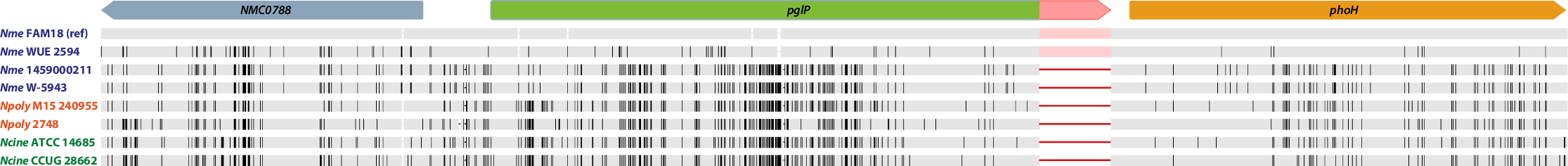

Supplement: S9 Fig — See Fig 7 and text for further details. (TIF) [file pgen.1008532.s009.tif]
