# Supplementary material for: Genetic determinants of genus-level glycan diversity in a bacterial protein glycosylation system
Source: PLoS Genet. 2019 Dec 23;15(12):e1008532. doi: 10.1371/journal.pgen.1008532 (PMC6959607; doi:10.1371/journal.pgen.1008532)
Supplement: S1 Table — (PDF) [file pgen.1008532.s010.pdf]

S1 Table. Lists of strains and plasmids used in this study

Strain name	Relevant genotype	Parent	Reference
KS100 (*N. gonorrhoeae* N400)	*recA6*	VD300	[50]
KS944	*N. elongata* subsp. *glycolytica* ATCC 29315		lab collection [62]
N. oralis F0314			lab collection
N. cinerea ATCC 14685			lab collection
KS992	*nirK-His::cat*	KS944	[25]
KS994	*nirK-His::cat pglC::kan*	KS992	[25]
KS1032	*nirK-His::cat pglP::kan*	KS992	This study
NK2259	Sm^r	KS944	This study
NW37	*nirK-His::cat* Sm^r	NK2259	This study
NW154	*pglP::kan/rpsL+*	NW37	This study
NW180	$pglP_{S74\text{-}R148del}$	NW154	This study
NW270	*NELON_11105::kan*	NW37	This study
NW182	$pglP_{N.\ oralis}$	NW154	This study
NW212	$pglP_{N.\ cinerea}$	NW154	This study
NW254	*pglP* rescue	NW154	This study
**Plasmid name**			
pAK220	*NELON_11110*	pCR2.1 TOPO	This study
pAK227	*NELON_11110::kan*	pAK220	This study
pKP79	pFLOB4300 *ermC'::kan/rpsL+*	pFLOB4300	This study
pFLOB4300	*ermC'/rpsL+*		[51]
