# Supplementary material for: Genetic determinants of genus-level glycan diversity in a bacterial protein glycosylation system
Source: PLoS Genet. 2019 Dec 23;15(12):e1008532. doi: 10.1371/journal.pgen.1008532 (PMC6959607; doi:10.1371/journal.pgen.1008532)
Supplement: S2 Table — (PDF) [file pgen.1008532.s011.pdf]

S2 Table

PCR oligonucleotide primers

Primer name	Sequence (5'-3')
av2934	GGTCGCCGATGGTGTTCAAGTGG
av2935	GCAGCAGGAATACGCGCCGGTTC
nw92	CAGACGGCCTGAAGTACG
nw94	GAAATTGTTATCCGCTTATCGGTTATTCCTTGTTTTCAG
nw95	CAAGGAATAACCGATAAGCGGATAACAATTTCACACAGG
nw96	CTGAAAGCTTTATCCCAGTCACGACGTTGTAAAAC
nw97	CGTCGTGACTGGGATAAAGCTTTCAGGCGGCC
nw98	GAGGAATACGATGCTGAAC
nw111	TAAAACATGCATTATCGGTTATTCCTTGTTTTC
nw112	GAATAACCGATAATGCATGTTTTAATCATCCC
nw113	GAAAGCTTTATCTAACTTTCATGGGTTCCTCCGAGTATTG
nw114	CATGAAAGTTAGATAAAGCTTTCAGGCGGC
nw122	CCGGTGAAATAGATAAAGCTTTCAGGCGGC
nw152	CGGTTCGGACATTATCGGTTATTCCTTGTTTTC
nw153	CCTGATTAAGCTGTGGCGCGTATAGGTGG
nw154	GCGCCACAGCTTAATCAGGCCGTGGCAG
nw180	CGGCATTCAAATAAGTGAG
nw181	GATGTTACATTGCACGCTACGGCTTTGAGACA
nw184	CGGTATCGATAAGCGCTGTATTGAAACGGCAG
nw185	GCACCACATAATTCAGCA
nw188	TCAAAGCCGTAGC CTCTGATGTTACATTGCAC
nw189	GCCGTTTCAATACAGCCGACGGTATCGATAAGC
