# Supplementary material for: Genetic determinants of genus-level glycan diversity in a bacterial protein glycosylation system
Source: PLoS Genet. 2019 Dec 23;15(12):e1008532. doi: 10.1371/journal.pgen.1008532 (PMC6959607; doi:10.1371/journal.pgen.1008532)
Supplement: S3 Table — (PDF) [file pgen.1008532.s012.pdf]

S3 Table

Pgl protein / gene sequences used in iterative BLASTP and BLASTN queries

pgl gene	function*	*N. gonorrhoeae* (FA1090)	*N. elongata sp. glycolytica* (ATCC 29315)	PubMLST locus designation
pglF	und-PP-glycan flippase	Q5FAD8 ngo0088	D4DSY1 Nelon_11115	NEIS0402
pglG	glycosyltransferase	Q5FAD9 ngo0087	D4DNG4 Nelon_10535	NEIS0401
pglH	glycosyltransferase	Q5FAE0 ngo0086	A0A0B5CSK6 Nelon_10545	NEIS0400
pglB	N-acetyltransferase / phosphoglycosyl transferase	Q5FAE1 ngo0085	D4DQH0/D4DQH1# Nelon_10565/ Nelon_10570#	NEIS2839/ NEIS2840
pglC	aminotransferase	Q5FAE2 ngo0084	D4DQH2 Nelon_10575	NEIS0379
pglD	NAD+-dependent dehydratase	Q5FAE3 ngo0083	A0A0B5CN10 Nelon_02985	NEIS0396
pglA	glycosyltransferase	Q5F602 ngo1765	-	NEIS0213
pglE	glycosyltransferase	Q5FA27 ngo0207	-	NEIS0568
pglP	glycosyltransferase	pseudogene	D4DSY2 Nelon_11110	NEIS2841
pglJ	UDP-GlcNAc(3NAc)A biosynthesis – 1st step C6-oxidase	-	A0A0B5CJR7 Nelon_10580	NEIS2842
pglK	UDP-GlcNAc(3NAc)A biosynthesis – 2nd step C3-dehydrogenase	-	D4DQH5 Nelon_10585	NEIS2843
pglM	UDP-GlcNAc(3NAc)A biosynthesis – 3rd step aminotransferase	-	A0A0B5CHH6 Nelon_05890	NEIS2844
pglN	UDP-GlcNAc(3NAc)A biosynthesis – 4th step acetyltransferase	-	D4DNJ5 Nelon_05885	NEIS2845
pglB2	glyceramidotransferase / phosphoglycosyl transferase	Q93TW2 *N. meningitidis* 053442	-	NEIS2838
Δ*pglG/H*	conserved deletion *pglG* 3´/*pglH* 5´	NA	NA	NEIS2473

- gene not present
* see Fig. 2 for details
# two (split) - ORF configuration
