# Supplementary material for: Genetic determinants of genus-level glycan diversity in a bacterial protein glycosylation system
Source: PLoS Genet. 2019 Dec 23;15(12):e1008532. doi: 10.1371/journal.pgen.1008532 (PMC6959607; doi:10.1371/journal.pgen.1008532)
Supplement: S1 Text — (PDF) [file pgen.1008532.s013.pdf]

## Oligonucleotide Sequences used to Assess *pglP* Pseudogene Mutation Distribution

*N. gonorrhoeae pglP* ORF-disrupting polymorphisms / SNVs (coding strand):

fs84 ( TTTCTT***CCCC***GCTTTCCGTTTATC ) - insertion of C (3C to 4C) relative to WT

ns207 ( CAAACATTTGAA***T***AAAAAGAAAA ) - replacement of A by T (stop codon generated)

fs387 ( TTCCGTCAGGCG***GCGC***GGAACACGG ) - insertion of GC relative to WT

*N. meningitidis pglP* ORF-disrupting polymorphisms / SNVs and CREE insertions (coding strand):

fs25 ( ACGGGA***TTTTTTT***CAAAAT ) - deletion of T (8T to 7T) relative to WT

fs60 ( CCTGACCGG***CC***TTACGGTTT ) - deletion of C (3C to 2C) relative to WT

CREE67 ( GACCCTTACGGTTTTGCAAA***ATATAGTGGATTAA*** ) - CREE sequence + AT

fs195 ( ATGGCAGTACCT***CCCCC***AACATA ) - insertion of C (4C to 5C) relative to WT

fs211 ( CAAAGAG***AAAAAAAA***CAACAAACCG ) - insertion of A (7A to 8A) relative to WT

CREEstop ( GCAATCGCAGAATACGGTAAGAAAATACCGGTGAA***ATATAGTGGATTAACAAAAATCAGGACAAGGCAAC*** ) – CREE sequence + AT
